# Supplementary material for: Cancer genome datamining and functional genetic analysis implicate mechanisms of ATM/ATR dysfunction underpinning carcinogenesis
Source: Commun Biol. 2021 Mar 19;4:363. doi: 10.1038/s42003-021-01884-x (PMC7979806; doi:10.1038/s42003-021-01884-x)
Supplement: Supplementary file 2 — Supplementary Information [file 42003_2021_1884_MOESM2_ESM.pdf]

Supplementary Materials for

Cancer genome datamining and functional genetic analysis implicate mechanisms of ATM/ATR dysfunction underpinning carcinogenesis

Erik Waskiewicz, Michalis Vasiliou, Isaac Corcoles-Saez and Rita S. Cha*

Correspondence to: r.cha@bangor.ac.uk

This PDF file includes:

- Supplementary Methods
- Supplementary Figure 1. Comparative analysis of different sequence- and structure-based alignment programs
- Supplementary Figure 2. Tissue specificity of the conserved ATM/ATR residues mutated in cancer
- Supplementary Figure 3. 3D mapping analysis of conserved ATM residues mutated in cancer in two different ATM models and a Tel1 model.
- Supplementary Figure 4. Molecular modelling analysis of ATM/ATR kinase domain residues mutated in cancer.
- Supplementary Figure 5. Genetic screen for isolating HU/MMS sensitive alleles of *MEC1*
- Supplementary Figure 6. Molecular modelling analysis of Mec1 residues critical for the DDR
- Supplementary Figure 7. Molecular modelling analysis of ATR and ATM residues corresponding to the Mec1 residues critical for the DDR.
- Supplementary Table 1. Nature of ATM and ATR alterations found in cancer
- Supplementary Table 2. DNA sequencing results of *mec1* alleles
- Supplementary References

SUPPLEMENTARY METHODS

Comparison of different sequence- and structure-based alignment tools

For ATM, ATR, Mec1 and Tel1 sequence alignment, we compared four different multiple sequence alignment (MSA) programs: Clustal Omega (https://www.ebi.ac.uk/Tools/msa/clustalo/), MUSCLE (https://www.ebi.ac.uk/Tools/msa/muscle/), MAFFT (https://www.ebi.ac.uk/Tools/msa/mafft/), and T-COFFEE (https://www.ebi.ac.uk/Tools/msa/tcoffee/). FASTA sequences of the respective proteins (Supplementary Figure 1a) were used for the analyses via the links provided above. All programs were run on EMBL-EBI servers using the default setting. The "Percent Identify Matrix" file from the "Results Summary" was downloaded by clicking on the relevant tab. Analysis shows small program-to-program variations; for example, the sequence identity between ATR ad Mec1 ranges from 22.0% (MUSCLE) to 27.8% (MAFFT), while the identify between ATM and Mec1 ranges from 18.3% (Clustal-Omega) to 21.8% (T-COFFEE)(Supplementary Figure 1b). The average percent identify was calculated by adding up each pairwise value and dividing by the total number of values (n=6). The value was lowest for MUSCLE (19.29%), followed by Clustal Omega (20.65%), MAFFIT (21.83%) and T-COFFEE (21.55%) (Supplementary Figure S1b). Overall, the average percent identities were comparable at around 20% average.

Evidence suggests that structure-based alignment programs might generate more reliable alignments than the sequence-based programs, especially for sequences sharing low level identity [1]. To compare efficacy, we utilized MATRAS (http://strcomp.protein.osaka-u.ac.jp/matras/cgi-bin/MulMat.cgi), a structure-based alignment program to align Mec1, ATR, ATM, and Tel1. We chose MATRAS because it is one of the top-performing structure-based alignment program available online[1]. The pairwise % identities among the four proteins and the average % identify value, 13.6% (Supplementary Figure 1c) are notably less than each of the sequence-based alignment programs examined (Supplementary Figure 1b). We infer that for the purpose of aligning Mec1, ATR, ATM, and Tel1, sequence-based programs would be more informative. Accordingly, we utilized Clustal Omega to align Mec1, ATR, ATM, and Tel1 (Supplementary Data 6). We selected Clustal-Omega because EMBL-EBI recommends this for protein alignments and MUSCLE or MAFFT for DNA alignments (https://www.ebi.ac.uk/Tools/msa/clustalw2/). FASTA file-links to Mec1, ATR, ATM, and Tel1 are provided in Supplementary Figure 1a. The UniProt IDs and FASTA file-links for ATRIP and Ddc2/Lcd1, respectively, are: Q8WXE1 (https://www.uniprot.org/uniprot/Q8WXE1.fasta) and Q04377 (https://www.uniprot.org/uniprot/Q04377.fasta).

DNA sequencing analysis

The *ARS/CEN/ADE2-mec1* plasmid from 50 transformants exhibiting strong sensitivity to MMS and/or HU were isolated and subjected to DNA sequencing analysis. All sequencing analysis was performed by Eurofins Genomics (Ebersberg, Germany) using the Value Read service giving typical reads of around 800 bases. Samples were sent pre-mixed; 15 µl of miniprep plasmid DNA (100 ng/µl) and 2 µl of the relevant sequencing primer (10 pmol) per reaction, either in 1.5ml Eppendorf tubes or in 96- well plate format. Primers were designed at regular intervals across the *MEC1* gene, 10 forward and 10 reverse (Supplementary Figure 5b) and were synthesized by Eurofins Genomics (Ebersberg, Germany). Results were aligned to the full *MEC1* gene sequence using the software CLC Main Workbench (Version 7.6.4) (Aarhus, Denmark). The quality of the sequencing reads was assessed by analysing the chromatograms in the .abi files and any poor quality sequence at the ends of the reads was trimmed off (Supplementary Data 5).

Supplementary Figure 1. Comparative analysis of different sequence- and structure-based alignment programs

a

Protein	UniProt ID	FASTA link
Mec1	P38111 / ATR_YEAST	https://www.uniprot.org/uniprot/P38111.fasta
Tel1	P38110 / ATM_YEAST	https://www.uniprot.org/uniprot/P38110.fasta
ATM	Q13315 / ATM_HUMAN	https://www.uniprot.org/uniprot/Q13315.fasta
ATR	Q13535 / ATR_HUMAN	https://www.uniprot.org/uniprot/Q13535.fasta

b

MUSCLE - https://www.ebi.ac.uk/Tools/msa/muscle/

	Mec1	ATR	ATM	Tel1
**Mec1**	100	21.96	18.46	18.77
**ATR**		100	19.62	18.24
**ATM**			100	18.71
**Tel1**				100

19.29% identity average

Clustal Omega - https://www.ebi.ac.uk/Tools/msa/clustalo/

	Mec1	ATR	ATM	Tel1
**Mec1**	100	23.58	18.27	18.84
**ATR**		100	21.24	18.84
**ATM**			100	22.61
**Tel1**				100

20.65% identity average

MAFFT - https://www.ebi.ac.uk/Tools/msa/mafft/

	Mec1	ATR	ATM	Tel1
**Mec1**	100	27.79	19.85	19.59
**ATR**		100	21.11	20.23
**ATM**			100	22.40
**Tel1**				100

21.83% identity average

T-COFFEE - https://www.ebi.ac.uk/Tools/msa/tcoffee/

	Mec1	ATR	ATM	Tel1
**Mec1**	100	23.00	21.82	20.13
**ATR**		100	21.83	19.56
**ATM**			100	22.97
**Tel1**				100

21.55% identity average

c

Protein	PDB ID
Mec1	5X6O
ATR	5YZ0
ATM	5NPO
Tel1	6JXC

MATRAS - http://strcomp.protein.osaka-u.ac.jp/matras/cgi-bin/MulMat.cgi

	Mec1	ATR	ATM	Tel1
**Mec1**	100	13.0	13.0	13.2
**ATR**		100	13.6	14.1
**ATM**			100	15.2
**Tel1**				100

13.6% identify average

a. The UniProt ID and FASTA link for the indicated protein sequences used for multiple sequence alignment (MSA) programs.
b. Pairwise percent identify matrix generated from the indicated MSA program. The highest and lowest values for each pairwise comparison are shown in green and red, respectively. For example, the identify between Mec1 and ATR was highest when aligned on MAFFT (27.78%) and lowest on MUSCLE (21.96%). "Identify average" was calculated by adding up each pairwise value and dividing by the total number of values (n=6).
c. Left panel: The PBD ID of the indicated protein structure used for structure-based alignment[2–5]. Right panel: Pairwise percent identity matrix generated from the MATRAS structure-based alignment program [1]. "Identity average" was calculated by adding up each pairwise value and dividing by the total number of values (n=6).

Supplementary Figure 2. Tissue specificity of the conserved ATM/ATR residues mutated in cancer

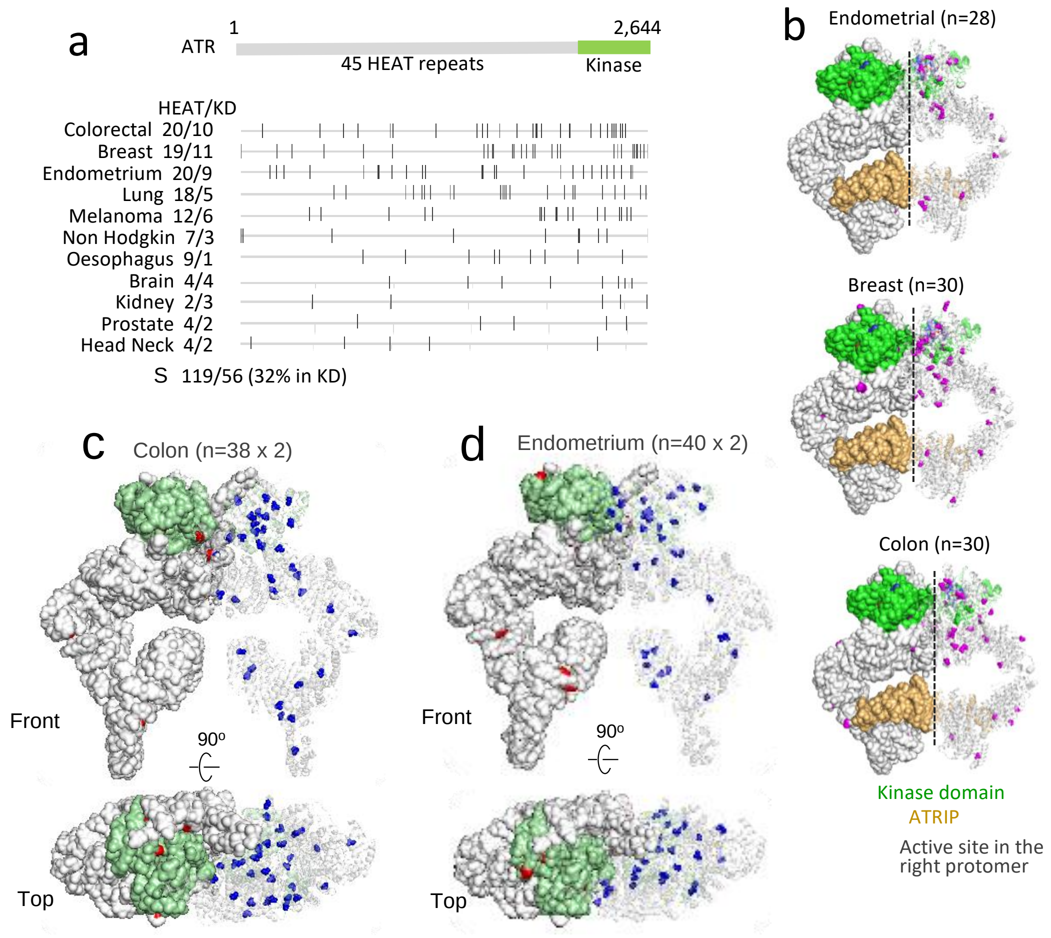

a. Locations of the conserved residues mutated in the indicated cancer type along the ATR polypeptide (Supplementary Data 4). "HEAT/KD": the number of residues in the respective regions. Overall, 32% (56/175) are in the kinase domain.

b. Conserved ATR residues mutated in endometrial (n=24), colorectal (n=27), and breast (n=27) cancers (Figure 2c; Supplementary Data 4) are mapped onto a cryo-EM model of the ATR-ATRIP enzyme complex (PDB 5YZ0, 4.70 Å)[2]. The mutated residues are shown as magenta spheres. The protomer on the right-hand side is in cartoon-representation and allows visualization of all mutated residues. The protomer on the left-side is shown in surface-representation to identify residues that are readily visible on the surface. Green: kinase domain. Brown: ATRIP. Pink circle: active site in the right protomer.

c. Conserved ATM residues mutated in colon- or endometrial-cancer are mapped onto a cryo-EM model of the dimeric ATM-complex (PBD 5NP0, 5.70 Å) [4]. The mutated residues in the left- and the right- protomers are shown in red and blue, respectively. The protomer on the right-hand side is in cartoon-representation and allows visualization of all mutated residues. The protomer on the left-side is shown in surface-representation to identify residues that are readily visible on the surface. Green: kinase domain. Upper- and lower-panels show a view from the front and the top, respectively.

Supplementary Figure 3. Mapping analysis of the conserved ATM residues mutated in cancer using two different ATM models and a Tel1 model.

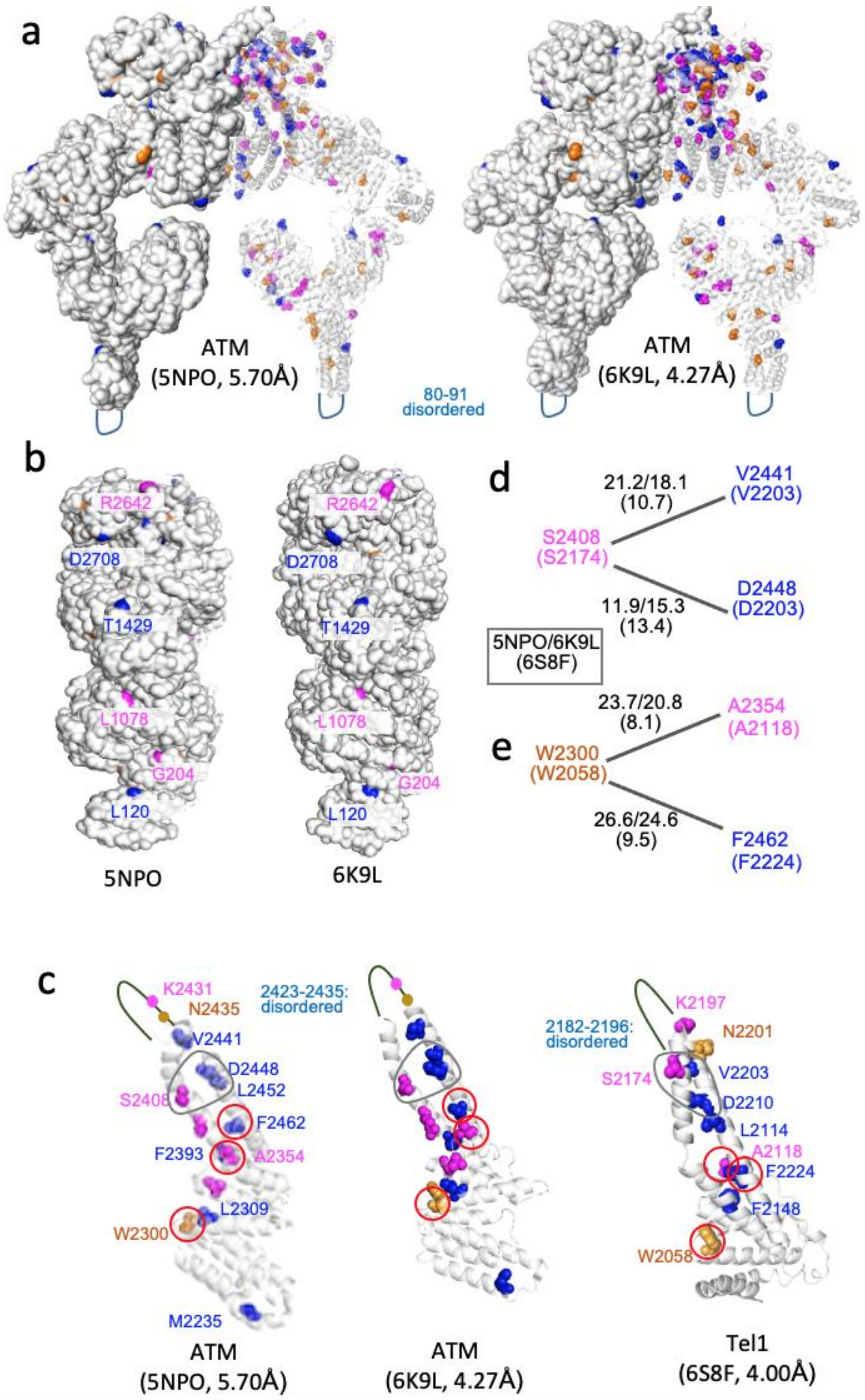

a. ATM residues mutated in endometrial (magenta), colorectal (orange), and non-Hodgkin's (blue) cancers are mapped onto two different ATM models; PBD 5NP0, 5.70 Å[4] and PBD 6K9L 4.27 Å [6]. The protomer on the right-hand side is in cartoon-representation and allows visualization of all mutated residues. The protomer on the left-hand side is shown in surface-representation to identify residues that are readily visible on the surface.
b. Side of views of the models in panel **a** showing the mutated residues that are readily visible on the surface.
c. High resolution images of the TRD3 in the two ATM models and a Tel1 model. The corresponding Tel1 residues are as indicated. Grey enclosed area: Distances between the three conserved residues are measured

and shown in panel **d.** Red circled residues: Distances between the three conserved residues are measured and shown in panel **e.**

d, e. Distances between the indicated residues in Å (see panel **c**). For each pair of residues, the two top numbers correspond to the distances measured in the ATM model 5NPO and 6K9L, respectively; the number in parenthesis underneath corresponds to the distance measured in the Tel1 model. The residue in parenthesis corresponds to the conserved Tel1 residue. In general, the distances measured in the two ATM models are closer than those measured in the Tel1 model. Note that the variations in the distances between the two ATM models are within the limit of resolution of the higher resolution model 6K9L of 4.27 Å.

Supplementary Figure 4. Molecular modeling analysis of ATM/ATR kinase domain residues mutated in cancer.

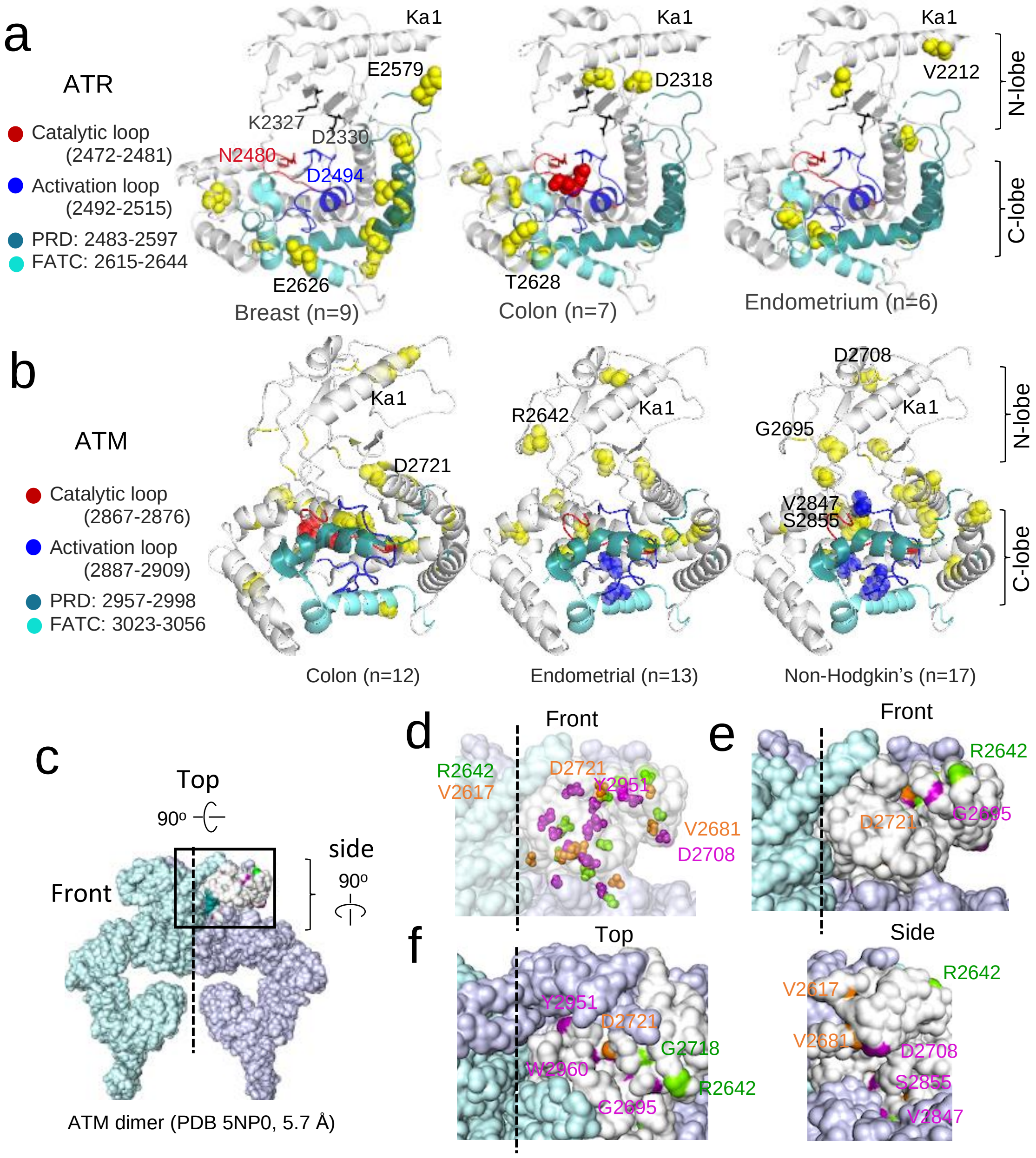

a. ATR-kinase domain showing location of the residues mutated in the indicated cancer. Labelled residues are solvent accessible (Figure 4d).

b. ATM-kinase domain showing location of the residues mutated in the indicated cancer. Labelled residues are solvent accessible (Figure 4f).

c. Surface representation of the ATM enzyme complex (PDB 5NPO, 5.70 Å)[4]. The dashed black line in the middle denotes the two fold symmetry axis. The left and right protomers are in white and blue, respectively. Black rectangle: area highlighted in panels **d** and **e.**

d. Higher resolution image of the area highlighted by a black rectangle in panel **c**. Magenta spheres: residues mutated in breast cancer shown in panel **d**. The transparency setting was "on" to visualize both the buried and exposed residues.

e. Same as in panel **d** except that the transparency setting was "off" to identify the solvent accessible residues.

f. A top- and side-views of the complex showing solvent accessible residues.

Supplementary Figure 5. Genetic screen for isolating HU/MMS sensitive alleles of *MEC1*

a

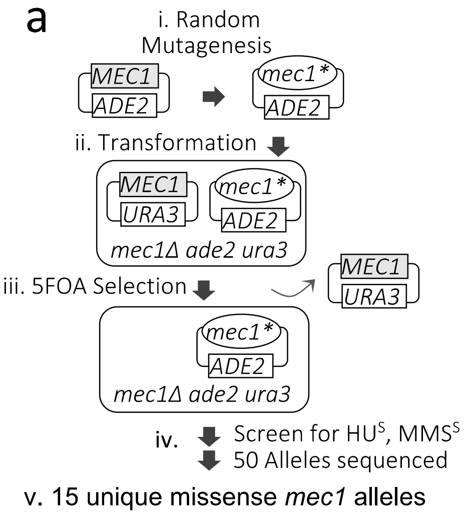

b

Primer	Sequence 5' – 3'	Location
F1	TGG AAT CAC ACG TCA AAT ATC	2
F2	CCA CAG AAT ATG GTT TAT GAG	351
F3	ATG CTT TGT AGA CCA CTT CCC	939
F4	GGA CTG TAA TTC TCG GAC GTC	1373
F5	GCT AAG ATT ATG TGT GAT GGC	2233
F6	GTG TAC AGT ATA CTG GAT ACC	2962
F7	TTA GGG AAG ATG ATG GCT CAC	3848
F8	TGC CAT CTC AAA CGA GGA TCC	4740
F9	AAT TTG CCC AAA GTT ATC ACG	5539
F10	ATC TCT TCG AGA AGA CTG TGG	6360
R1	GGC CAG CAA CAC TCA ATA CCA G	7006
R2	TCG AGT TTT CCA TCT ACT GCG	6495
R3	ATC TTC CGT AGC CTT ACT CAG	5620
R4	TTG TTC CGA TAA GCC TAA AGG	4790
R5	AAT ATC AGC GTA TGG AGT TCC	4925
R6	TCA CCA TTC TTG CAA ATT GGC	3080
R7	CCG TGT TTT ACC AAA GCT ACG	2319
R8	GGA TTT ATT CGC ACG AAA GGG	1500
R9	AAC CTT AGC TTT TCC AGC TGC	1173
R10	TCA AAG TTC CCG TTG GAA TCC	273

c

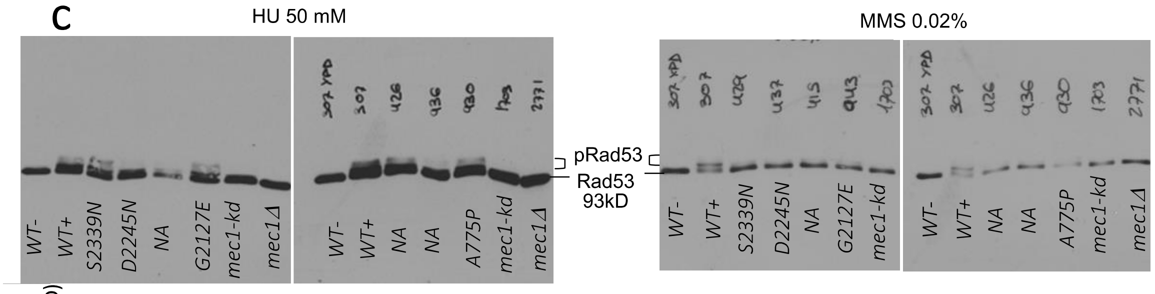

d

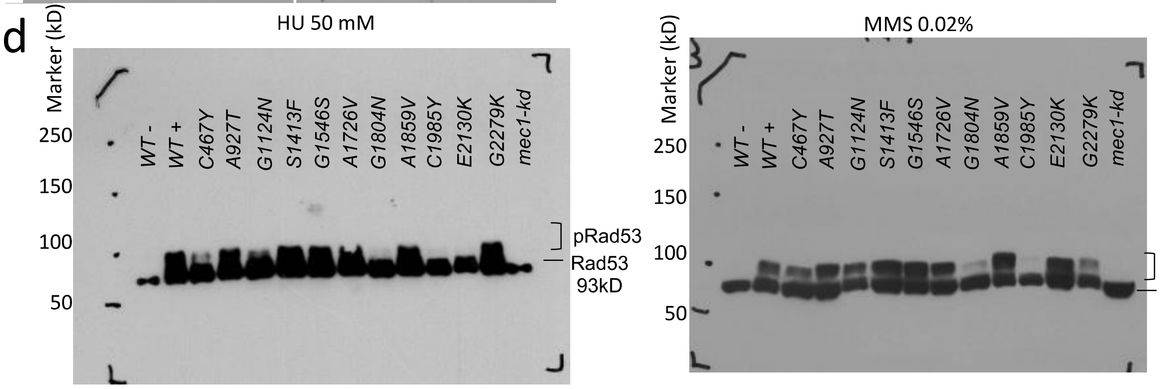

a. Schematic representation of the screen. (i) An *ARS-CEN-ADE2* plasmid containing the entire *MEC1* open reading frame was subjected to hydroxylamine random mutagenesis. (ii) A pool of mutagenized plasmids, each carrying a putative mutated allele ("*mec1**") was transformed into a *mec1Δ ade2 ura3* strain carrying an *ARS-CEN-URA* plasmid carrying a wild type copy of *MEC1*. This strain does not have any other mutation(s) in the genome. (iii) The transformants (i.e. *ADE* prototrophs) were replica plated onto 5 fluoroorotic acid (5FOA) plates to select against the *MEC1-URA3* plasmid. (iv) ~ 10,000 viable 5FOA resistant transformants were screened for sensitivity to HU and/or MMS. Approximately 200 strains showed strong sensitivity to either or both stresses.

Plasmids were isolated from 50 strains and subjected to DNA sequencing analysis. (v) 26 of the 50 were found to contain a mutation(s) leading to an amino acid change(s) (Supplementary Table 2; Supplementary Data 5).
b. Primers used for sequencing of *mec1* alleles. “F” and “R” denote forward and reverse direction primers, respectively. “Location” refers to the starting base of each primer in the *MEC1* DNA sequence. All primers were designed to give similar length and melting temperature. Primers were ordered from Eurofins Genomics (Ebersberg, Germany) using the SeqPrimer service, giving high quality primers for sequencing (UPLC purites of around 90%, checked by MALDI-TOF-MS).
c. Impact of the indicated *mec1* allele on HU- and MMS-dependent Rad53 activation. Western blot analysis was performed using a Rad53 antibody that detects both unphosphorylated and phosphorylated species (EL7.E1)[7]. “WT-“: untreated control sample. “*mec1-kd”:* a kinase dead mutant used as a control; the strain is in a *sml1Δ* background, necessary for viability[8,9]. The images represent a full-size uncropped Western blots. NA: Not Applicable, an irrelevant sample. The numbers above some samples are strain numbers used and can be ignored.
d. Uncropped full size images of the data presented in Figure 5b.

Supplementary Figure 6. Molecular modelling analysis of the 15 Mec1 residues critical for the DDR

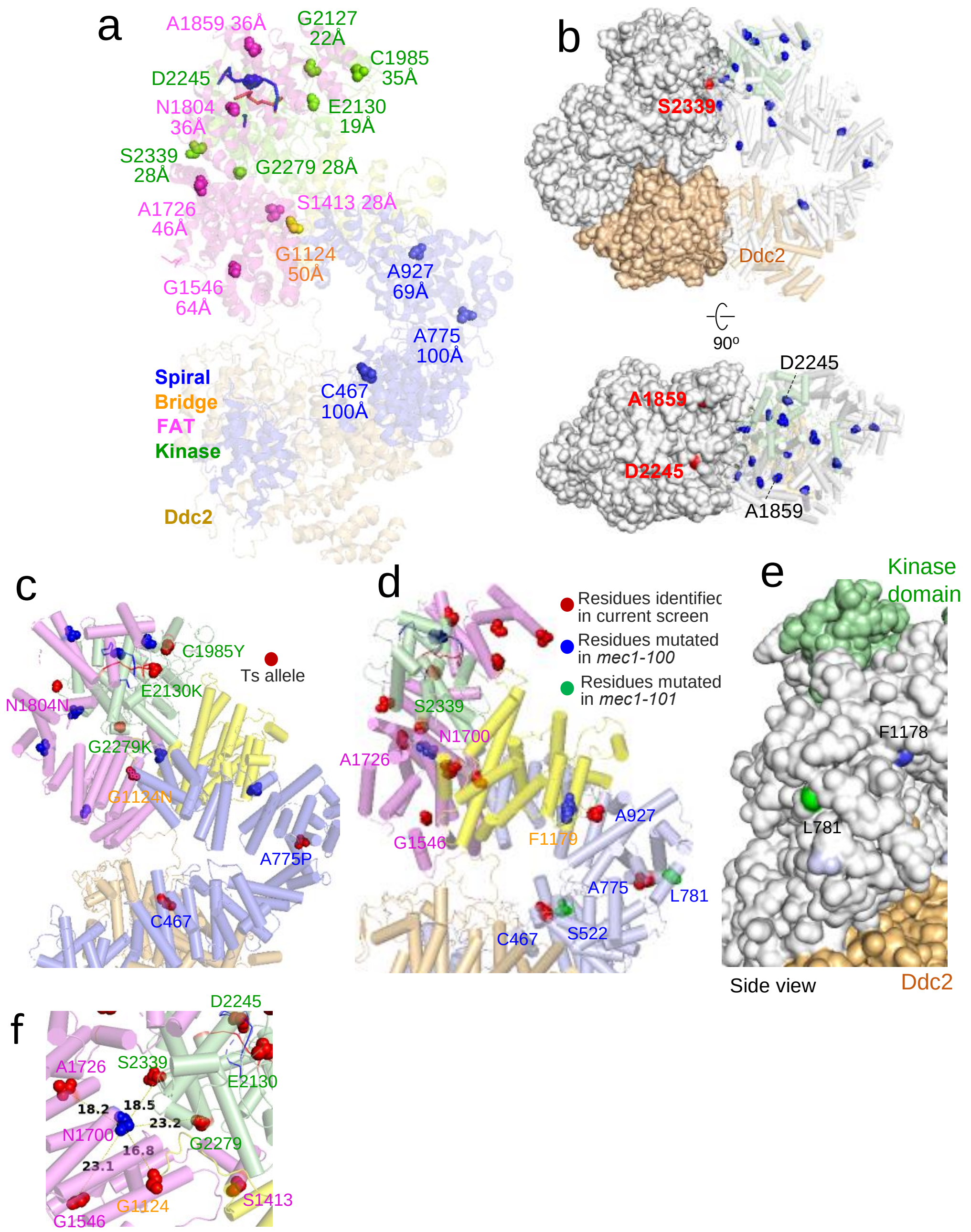

a. 15 Mec1 residues critical for the DDR are mapped onto a Mec1-Ddc2 protomer (PBD 5X60, 3.9 Å)[3]. The Mec1 polypeptide is shown in blue, yellow, violet, and green, representing the spiral, bridge, FAT, and kinase domains, respectively. The activation- and catalytic-loops are shown in blue and red, respectively. Only the D2245 is in the active site. Distance between D2245 and each residue is indicated.

b. Location of the 15 residues (panel **a**) in a cryo-EM model of the dimeric Mec1 – Ddc2 enzyme complex [3]. Each protomer comprise a Mec1 and Ddc2 polypeptides shown in white and brown, respectively. The right protomer is in cartoon representation and shows all 15 residues in blue. The left is in surface representation and shows only the solvent accessible residues in red.

c. Seven of the 15 *mec1* mutations confer temperature sensitivity (Figure 5d). The residue mutated in each of the seven temperature-sensitive allele is shown in red.

d. Co-localization of the residues identified in the current screen (red) and a previous unbiased screen, where two separation of function alleles, *mec1-100* and *mec1-101,* were isolated[8]. The residues mutated in *mec1-100* are shown in blue and those mutated in *mec1-101* are shown in green.

e. Two of the four residues mutated in *mec1-100* or *mec1-101* are solvent accessible.
f. The N1700 (blue residue) mutated in *mec1-101* are within ~20Å of the five residues (G1124, G1546, A1726, G2279, and S2339; red residues) identified in the current study.

Supplementary Figure 7. Molecular modeling analysis of conserved Mec1, ATR, and ATM residues

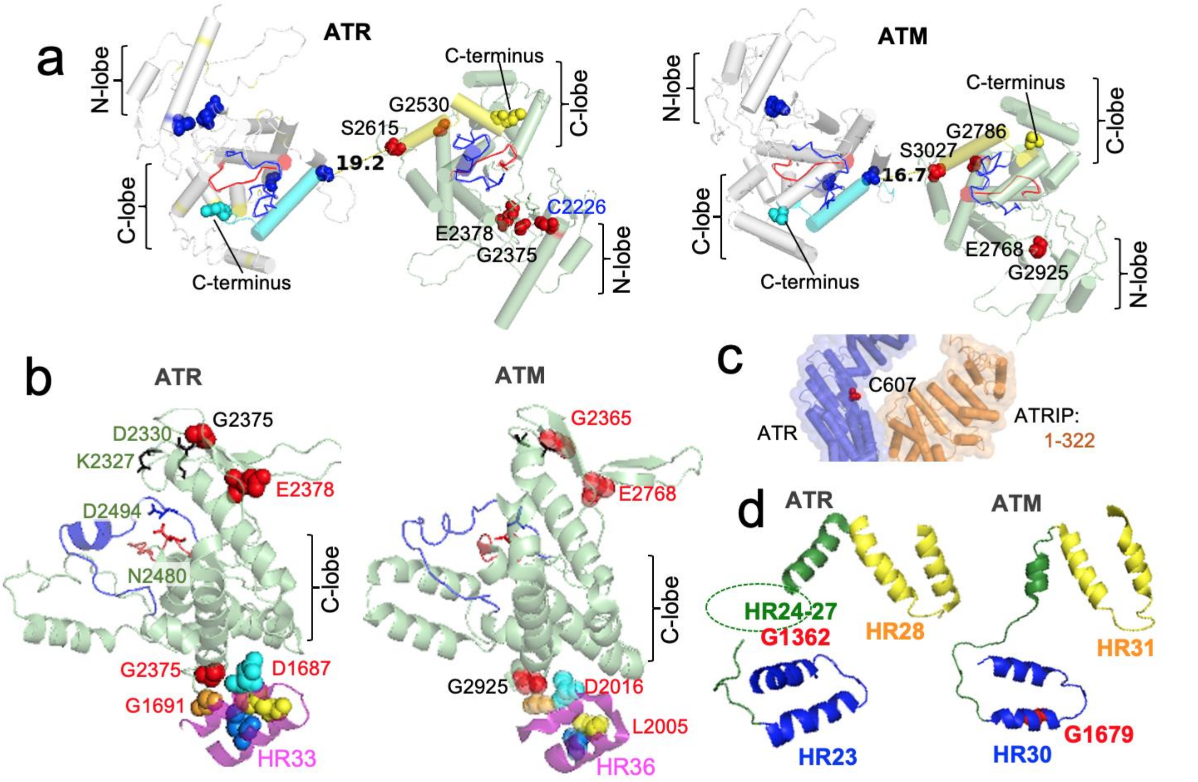

a. Top view of the dimeric ATR (left) and ATM (right) enzyme complexes showing only the kinase domains. The five conserved ATR residues and the four conserved ATM residues in the left and right protomers are shown in blue and red, respectively.

b. Kinase domain of ATR and ATM showing conserved residues. The ATR G2375 and ATM G2925 correspond to the ATM G2279. These residues are next to a HR unit in respective FAT domain located more than 800 residues away. The HR contains four absolutely conserved residues (Figure 6g). Red residue: mutated in cancer (Figure 5c; Supplementary Data 3 and 4). The green residues in ATR are involved in ATP binding (K2327, D2330) and Mg2+ stabilization (D2480, D2494)[2].

c. The ATR607 is likely to be at an interface between ATR and ATRIP. The published ATR-ATRIP model complex is missing residues 323-795[2].

d. The ATR G1362, corresponding to the Mec1 G1124 is in a flexible region between the spiral and bridge domains. The ATM G1679, corresponding to the Mec1 G1124 is in the last HR unit of the spiral domain connected to the linker preceding the bridge domain.

Supplementary Table 1. Nature of ATM and ATR alterations found in cancer

Source data used to create Figure 1e and 1f. The number of mutations in both ATM and ATR are split by cancer type. The number of patients and samples are given for each cancer type, and the mutations present are split by the type of mutation.

			ATM							ATR						
	a	b	c	d	e	f	g	h	i	c	d	e	f	g	h	i
**Cancer**	**# Patients**	**# Samples**	**# Mutated**	**% Mutated**	**% Missense Mutation**	**Missense**	**Trunc**	**In frame**	**Other**	**# Mutated**	**% Mutated**	**% Missense Mutation**	**Missense**	**Trunc**	**In frame**	**Other**
Endometrial/uterus	1790	1799	205	11	10.2	183	112	4	1	163	9	6.9	124	49	0	0
Bladder	2019	2066	217	11	9.5	180	64	4	1	129	6	4.5	96	9	0	0
Non Hodgkin	3190	3190	267	8	8.4	222	97	3	1	49	2	1.5	48	2	0	0
Colorectal	3396	3504	198	9	8.2	266	140	4	1	126	4	4	130	46	1	1
Oesophagus/Stomach	3052	3089	263	9	7.2	181	100	1	0	203	7	4.6	91	65	0	1
Melanoma	1410	1462	124	8	6.9	93	18	0	0	106	7	7	93	19	0	0
Lung (NSC)	5322	5606	420	7	6.7	280	148	2	2	455	8	3.6	188	46	0	1
Cervical	605	607	42	7	3.6	18	4	0	0	65	11	2.5	16	2	0	0
Prostate	4628	4850	257	5	3.4	106	66	3	0	153	3	0.7	26	8	1	0
Liver	1487	1507	55	4	3.2	280	148	2	2	42	3	2.2	28	9	0	1
Pancreatic	1206	1207	40	3	2.8	30	13	0	0	18	1	1	15	4	0	0
HeadNeck	1860	1862	66	4	2.4	47	2	0	0	182	10	3.6	65	5	0	0
Kidney	3452	3528	81	2	1.8	42	37	0	0	46	1	0.8	20	9	0	0
Breast	8806	9133	229	3	1.7	109	64	2	1	240	3	2	157	32	6	2
Ovarian	1680	1692	66	4	1.1	18	3	0	1	137	8	0.7	12	2	0	0
Brain	4931	5065	52	1	0.7	33	14	2	0	51	1	0.8	40	8	0	1
Testis	479	485	12	2	0.6	3	0	0	0	4	1	0.8	4	0	0	0
Meyloid	2224	2514	16	1	0.3	5	2	0	0	2	0	0	1	0	0	0
Neuroblastoma	1549	1472	7	0	0.3	3	1	0	0	0	0	0	0	0	0	0

For each cancer type, the following information was provided:

a,b. The number of patients and tumor samples
c. The number of samples carrying one or more of missense, truncation, in frame, or other genetic alterations.
d. The fraction of samples carrying one or more of the genetic alterations mentioned in c or c/b*100.

e. The fraction of samples carrying one or more of missense mutation(s) or f/b*100.

f. The number of samples carrying one or more of missense mutation(s).

g. The number of samples carrying a truncation mutation.

h. The number of samples carrying an in-frame mutation.

i. The number of samples carrying "other" types of mutation.

The numbers in column "e" was used for Figure 1e.

Supplementary Table 2. DNA sequencing results of *mec1* alleles

Of the 50 *mec1* alleles sequenced, 24 did not carry a mutation in *MEC1.* We suspect that the strains from which the latter plasmids were isolated carry an incidental mutation(s) elsewhere in the genome linked to the HU/MMS sensitivity. Two alleles (EW47 and 48) carry a truncation mutation at the 27th codon (Q27X); it is likely that the strains from which the plasmids were isolated carry a second site suppressor mutation. AA: amino acid; HR: HEAT repeat; IHR: Inter HR units. Bold and shaded: alleles and mutations examined in current study (Supplementary Data 5).

	Allele	Base change	Codon change	AA change	Region
1	mec1-EW1	C-T 2967	TAC-TAT	Silent	–
		C-T 5177	GCT-GTT	A1726V	HR37/FAT
		G-A 6303	CTG-CTA	Silent	–
		G-A 6305	AGT-AAT	S2102N	Kinase
2	**mec1-EW2**	C-T 1215	AAC-AAT	Silent	–
		G-A 3370, G-A 3371	GGT-AAT	**G1124N**	IHR25
3	**mec1-EW3**	C-T 5576	GCG-GTG	**A1859V**	IHR40
4	**mec1-EW4**	G-A 2454	AAG-AAA	Silent	–
		G-A 4636	GGC-AGC	**G1546S**	HR33
		G-A 5447	AGG-AGA	Silent	–
5	**mec1-EW5**	C-T 5177	GCT-GTT	**A1726V**	HR37/FAT
6	mec1-EW6	G-A 1763	AGT-AAT	R588N	HR13
		G-A 1769	AGA-AAA	R590K	HR13
		G-A 5467 G-A 5468	GGT-AAT	G1823N	IHR39/FAT
7	**mec1-EW7**	G-A 1400	TGT-TAT	**C467Y**	HR11
		C-T 5908	CTA-TTA	Silent	–
8	**mec1-EW10**	G-A 6835, G-A 6836	GGA-AAA	**G2279K**	Kinase
9	**mec1-EW15**	C-T 4238	TCT-TTT	**S1413F**	HR30
10	**mec1-EW21**	G-A 2779	GCC-ACC	**A927T**	HR21
		G-A 2784	CAG-CAA	Silent	–
11	mec1-EW23	C-T 4238	TCT-TTT	S1413F	HR30
12	**mec1-EW29**	G-A 5410, G-A 5411	GGC-AAC	**G1804N**	HR39/FAT
13	mec1-EW32	G-A 2779	GGC-ACC	A927T	HR21
		G-A2784	CAG-CAA	Silent	-
14	**mec1-EW39**	G-A 6388	GAA-AAA	**E2130K**	Kinase
15	**mec1-EW46**	G-A 3693	TTG-TTA	Silent	-
		G-A 5954	TGT-TAT	**C1985Y**	HR43
		C-T 5991	GGC-GGT	Silent	–
16	mec1-EW47	C-T 79	CAG-TAG	Q27X	HR1
17	mec1-EW48	C-T 79	CAG-TAG	Q27X	HR1
18	mec1-4	G-A 6388	GAA-AAA	E2130K	Kinase
19	**mec1-11**	G-C 2323	GCT-CCT	**A775P**	HR18
20	mec1-40	G-A 4637	GGC-GAC	G1546D	HR33
21	mec1-119	C-T 3917	GCA-GTA	A1306V	HR29
22	mec1-121	C-T 3659	TCC-TTC	S1220F	HR27
23	mec1-155	C-T 1967	TCT-TTT	S656F	HR15
		G-A 6724	GTA-ATA	V2242I	Kinase
24	**mec1-156**	G-A 6380	GGG-GAG	**G2127E**	Kinase
25	**mec1-219**	C-T 2337	CAC-CAT	Silent	–
		G-A 7014	AGT-AAT	Silent	–
		G-A 7016	TTG-TTA	**S2339N**	Kinase
26	**mec1-222**	C-T 4935	AAC-AAT	Silent	–
		G-A 6733	GAC-AAC	**D2245N**	Kinase

Supplementary References

1. Carpentier, M. & Chomilier, J. Protein multiple alignments: sequence-based versus structure-based programs. *Bioinformatics* **35**, 3970–3980 (2019).
2. Rao, Q. *et al.* Cryo-EM structure of human ATR-ATRIP complex. *Cell Res.* **28**, 143–156 (2018).
3. Wang, X. *et al.* 3.9 Å structure of the yeast Mec1-Ddc2 complex, a homolog of human ATR-ATRIP. *Science (80-.).* **358**, 1206–1209 (2017).
4. Baretic, D. *et al.* Structures of closed and open conformations of dimeric human ATM. *Sci. Adv.* **3**, e1700933 (2017).
5. Wang, X. *et al.* Structure of the intact ATM/Tel1 kinase. *Nat. Commun.* **7**, 11655 (2016).
6. Xiao, J. *et al.* Structural insights into the activation of ATM kinase. *Cell Research* 683–685 (2019). doi:10.1038/s41422-019-0205-0
7. Fiorani, S., Mimun, G., Caleca, L., Piccini, D. & Pellicioli, A. Characterization of the activation domain of the Rad53 checkpoint kinase. *Cell Cycle* **7**, 493–499 (2008).
8. Paciotti, V., Clerici, M., Scotti, M., Lucchini, G. & Longhese, M. P. Characterization of mec1 kinase-deficient mutants and of new hypomorphic mec1 alleles impairing subsets of the DNA damage response pathway. *Mol. Cell. Biol.* **21**, 3913–3925 (2001).
9. Zhao, X., Muller, E. G. . & Rothstein, R. A Suppressor of Two Essential Checkpoint Genes Identifies a Novel Protein that Negatively Affects dNTP Pools. *Mol. Cell* **2**, 329–340 (1998).
