# Supplementary material for: Cancer genome datamining and functional genetic analysis implicate mechanisms of ATM/ATR dysfunction underpinning carcinogenesis
Source: Commun Biol. 2021 Mar 19;4:363. doi: 10.1038/s42003-021-01884-x (PMC7979806; doi:10.1038/s42003-021-01884-x)
Supplement: Supplementary file 3 — Description of Additional Supplementary Files [file 42003_2021_1884_MOESM3_ESM.pdf]

Description of Additional Supplementary Files

File name: Supplementary Data 1.

Description: Missense mutations of ATM found in cancer. Source file for the "ATM" panel in Figure 1b. A tab-separated-value (tsv) file was downloaded from the cBio Cancer Genomics Portal Website (http://cbioportal.org). The file contains all ATM missense mutations identified in over 46,000 cancer samples in the data base.

File name: Supplementary Data 2.

Description: Missense mutations of ATR found in cancer. Source file for the "ATR" panel in Figure 1a. A tab-separated-value (tsv) file was downloaded from the cBio Cancer Genomics Portal Website (http://cbioportal.org). The file contains all ATR missense mutations identified in over 46,000 cancer samples in the data base.

File name: Supplementary Data 3.

Description: Conserved ATM residues mutated in cancer. ATM residues that are mutated in cancer and conserved in Mec1 and/or Tel1 are identified via manual analysis. Locations of the conserved residues in Mec1 and Tel1 are shown in black and red, respectively.

File name: Supplementary Data 4.

Description: Conserved ATR residues mutated in cancer. ATR residues that are mutated in cancer and conserved in Mec1 and/or Tel1 are identified via manual analysis. Locations of the conserved residues in Mec1 and Tel1 are shown in black and red, respectively.

File name: Supplementary Data 5.

Description: Sequencing results of mec1 alleles characterised in current study. Upper panel: Primer coverage across the whole of MEC1. Forward primers are coloured green and reverse primers are coloured red (Supplementary Figure 5b). Trimmed sequences due to poor quality sequencing are shown in light green/red. Lower panel: Chromatograms of each mutation identified and the outcome of that mutation. For each mutation, at least one forward- and one reverse- read covered the mutation.

File name: Supplementary Data 6.

Description: Clustal Omega multiple sequence alignment of Mec1, ATR, ATM, and Tel1. FASTA sequences of Mec1, ATR, ATM, and Tel1(Supplementary Figure 1a) were used as input for Clustal Omega alignment program (https://www.ebi.ac.uk/Tools/msa/clustalo/) (Supplementary Methods). The 15 Mec1 residues and the nature of mutations examined in current study are shown in red (Figure 5a).
