# Supplementary material for: Cancer genome datamining and functional genetic analysis implicate mechanisms of ATM/ATR dysfunction underpinning carcinogenesis
Source: Commun Biol. 2021 Mar 19;4:363. doi: 10.1038/s42003-021-01884-x (PMC7979806; doi:10.1038/s42003-021-01884-x)

FIGURE 1 – EW 1

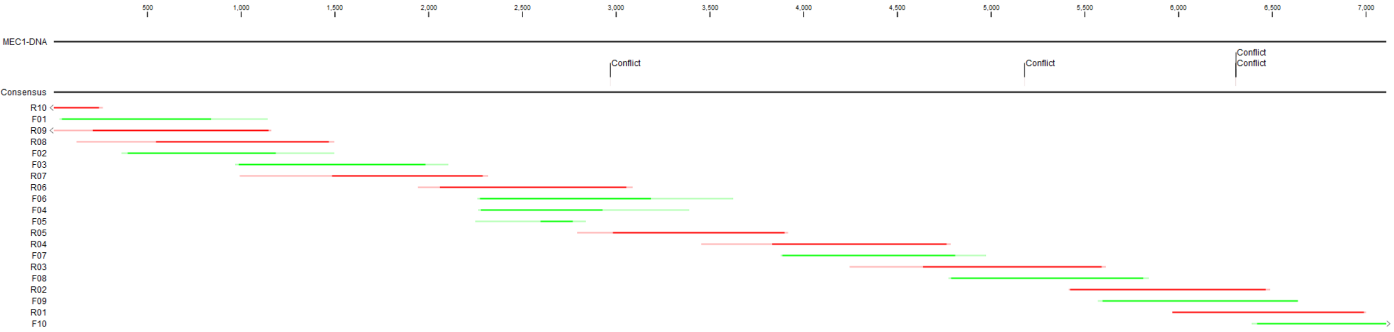

2967 C→T (silent)

5177 C→T (A1726V)

6303 G→A (silent)
6305 G→A (S2102N)

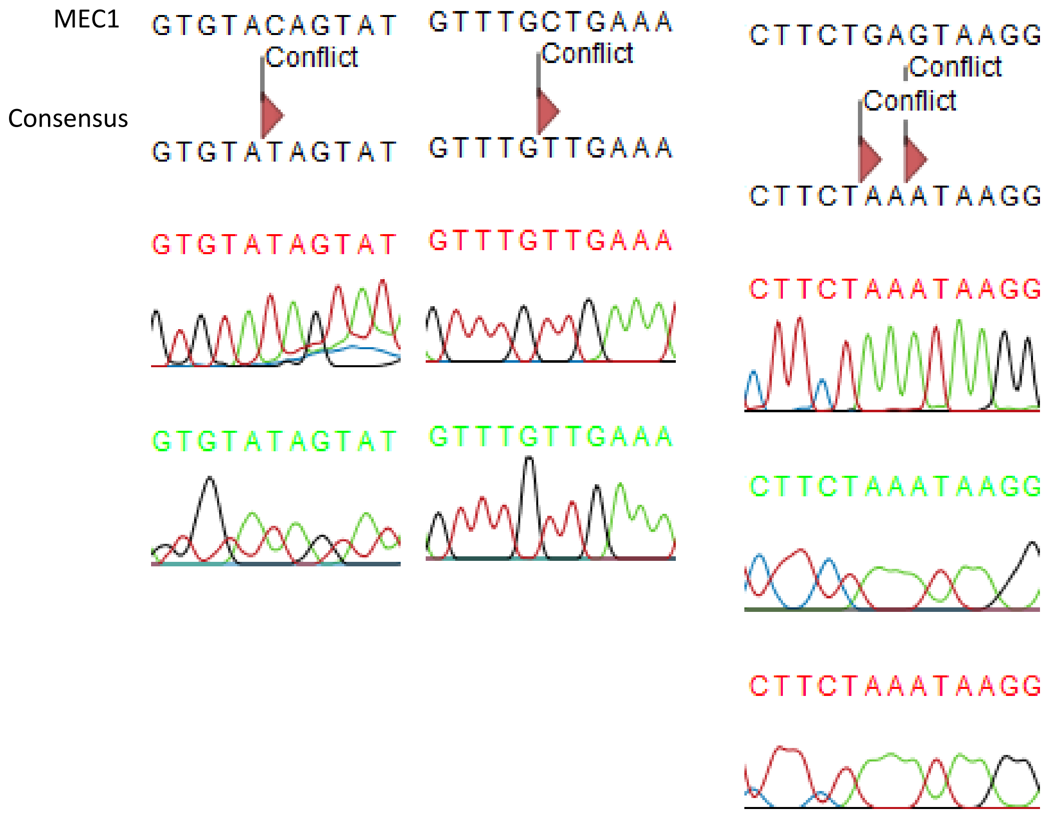

FIGURE 2 – EW 2

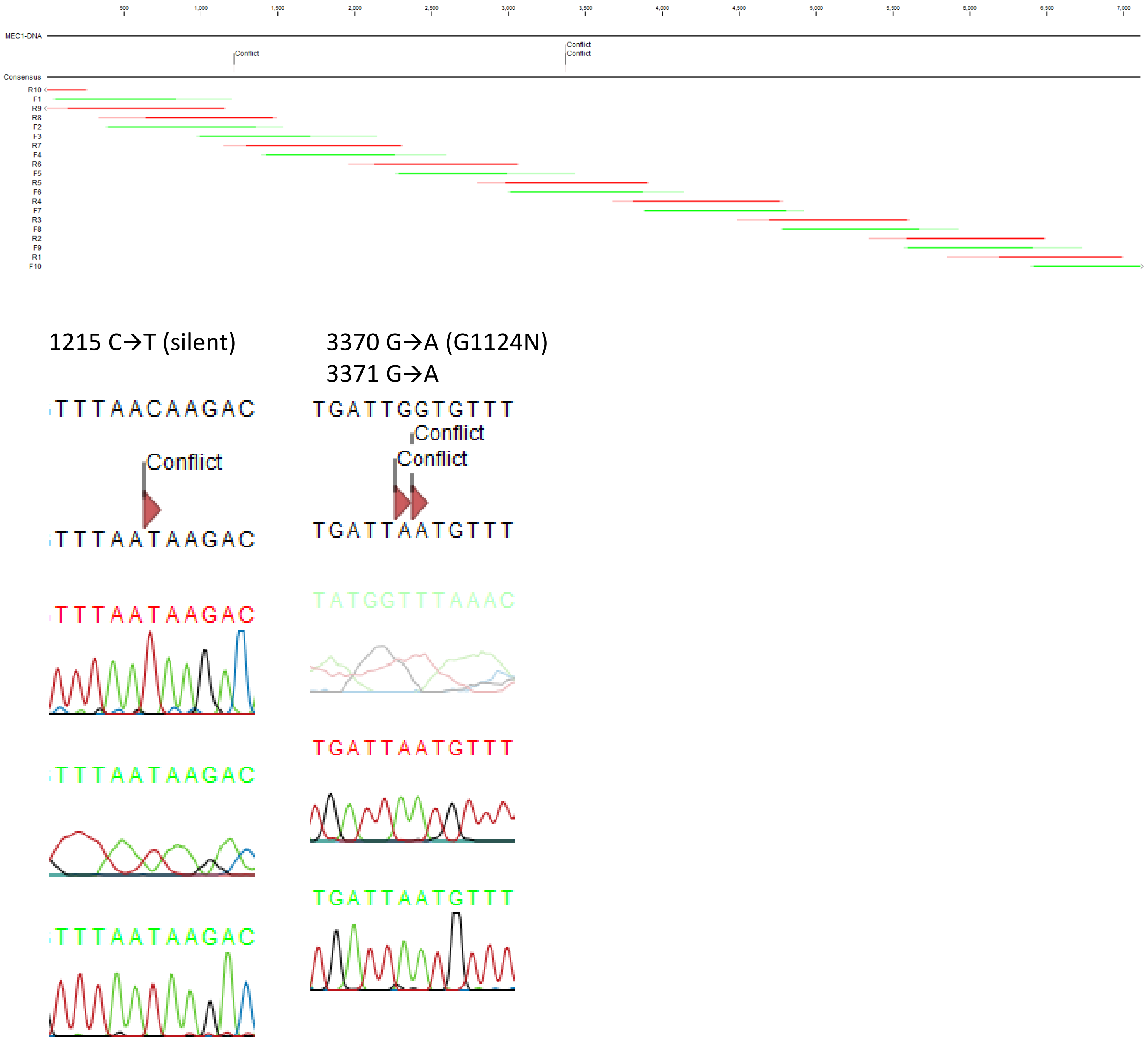

500
1,000
1,500
2,000
2,500
3,000
3,500
4,000
4,500
5,000
5,500
6,000
6,500
7,000
MEC1-DNA
Conflict
Conflict
Conflict
Consensus
R10
F1
R9
R8
F2
F3
R7
F4
R6
F5
R5
F6
R4
F7
R3
F8
R2
F9
R1
F10
1215 C→T (silent)
3370 G→A (G1124N)
3371 G→A
TTTAACAAGAC
TGATTGGTGTTT
Conflict
Conflict
Conflict
TTTAATAAGAC
TGATTAATGTTT
TTTAATAAGAC
TATGGTTTAAAC
TTTAATAAGAC
TGATTAATGTTT
TTTAATAAGAC
TGATTAATGTTT

FIGURE 3 – EW 3

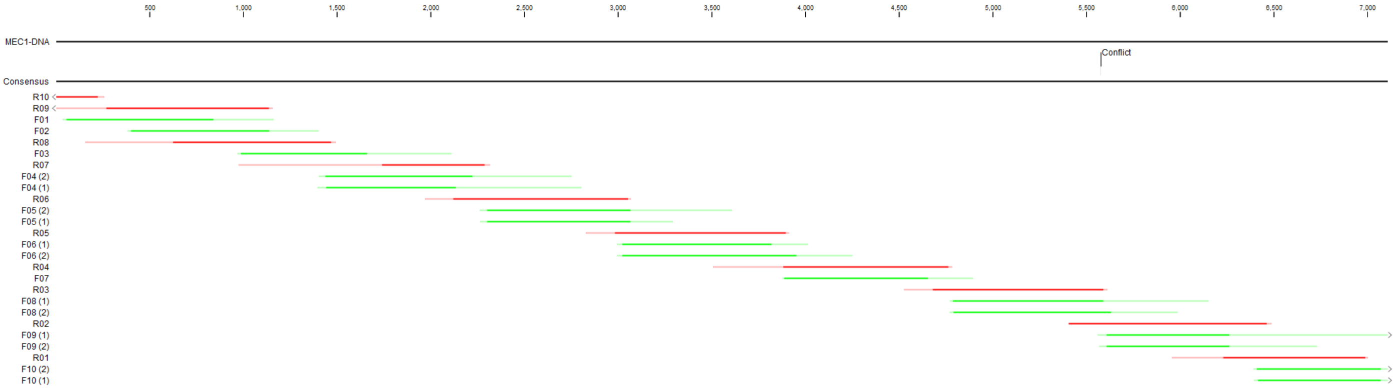

5576 C→T (G1124N)

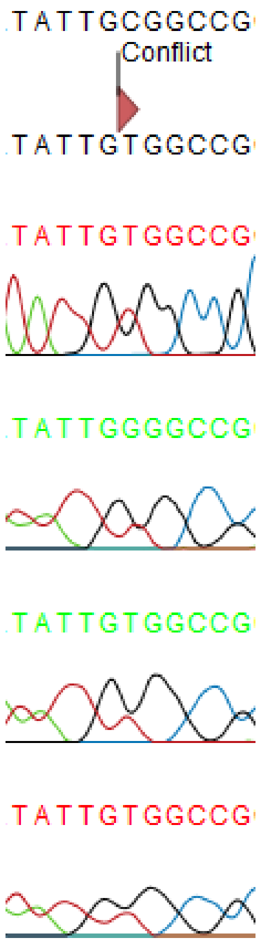

FIGURE 4 – EW 4

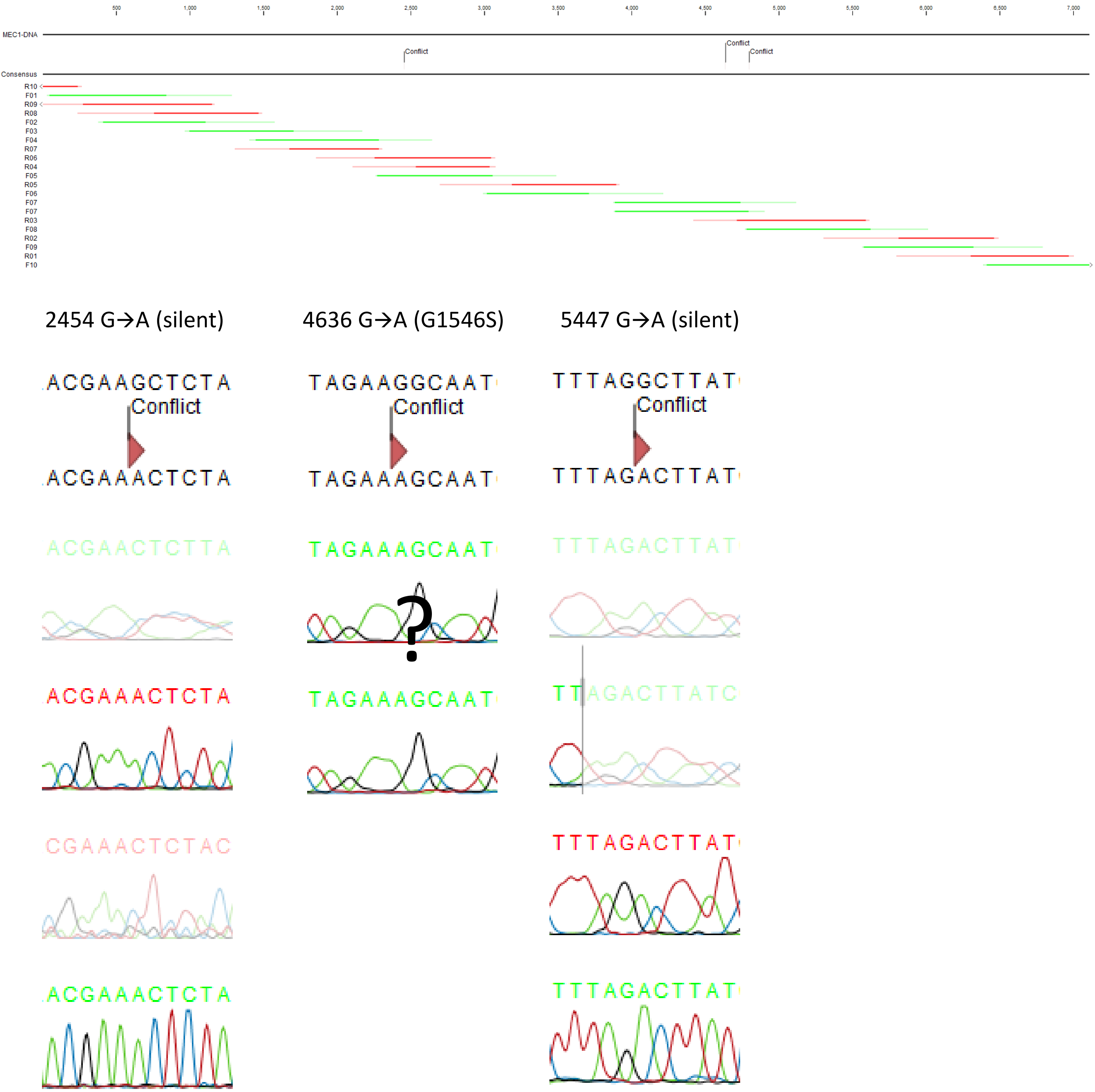
MEC1-DNA
Consensus
Conflict
Conflict
Conflict
R10
F01
R09
R08
F02
F03
F04
R07
R06
R04
F05
R05
F06
F07
F07
R03
F08
R02
F09
R01
F10
2454 G→A (silent)
4636 G→A (G1546S)
5447 G→A (silent)
ACGAAGCTCTA
Conflict
ACGAAACTCTA
TAGAAGGCAAT
Conflict
TAGAAAGCAAT
TTTAGGCTTAT
Conflict
TTTAGACTTAT
?

FIGURE 5 – EW 5 (missing R8)

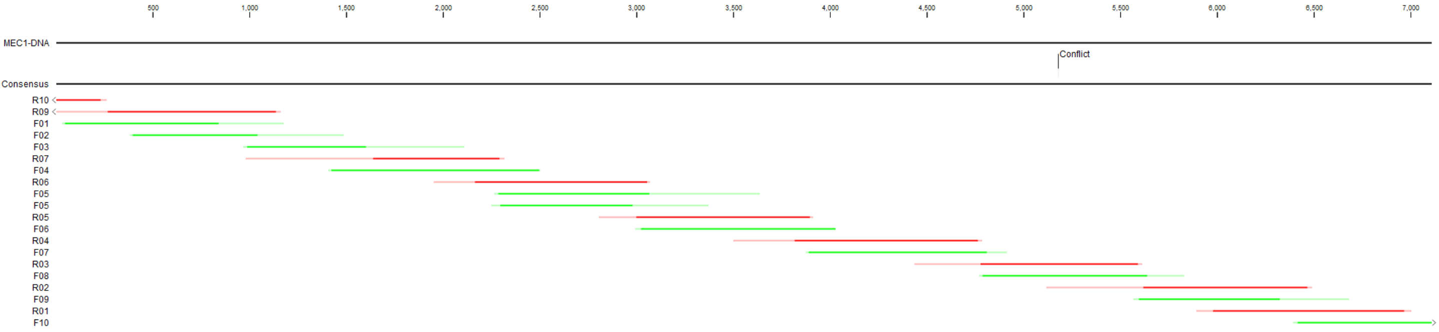

5177 C→T (A1726V)

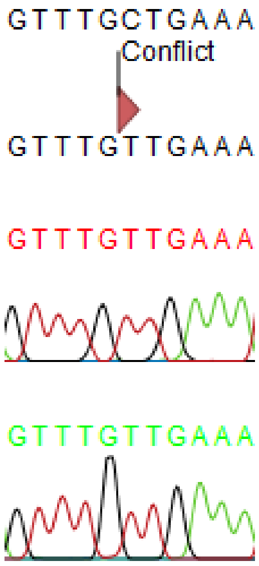

FIGURE 6 – EW 6

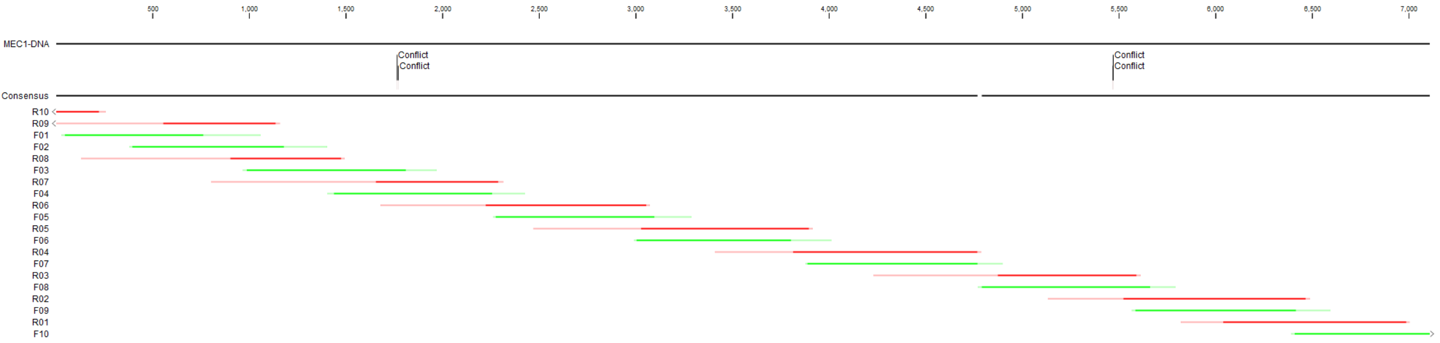

1763 G→A (S588N)
1769 G→A (R590K)

5467 G→A (G1823N)
5468 G→A

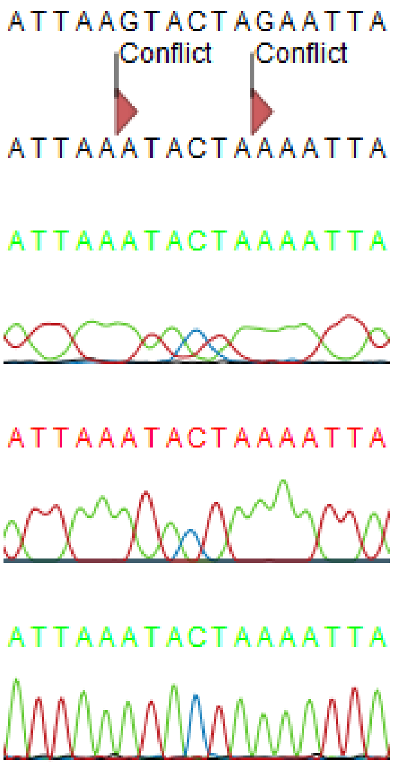

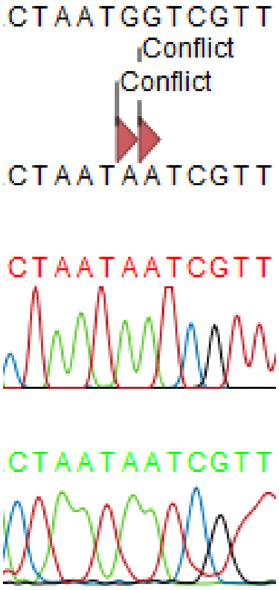

Poor coverage:

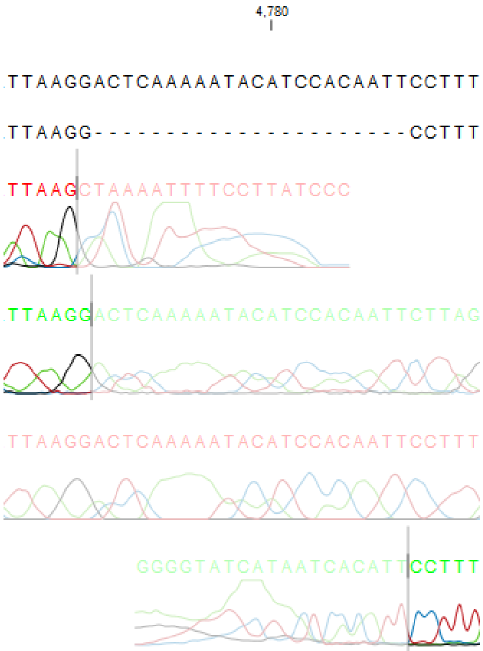

FIGURE 7 – EW 7

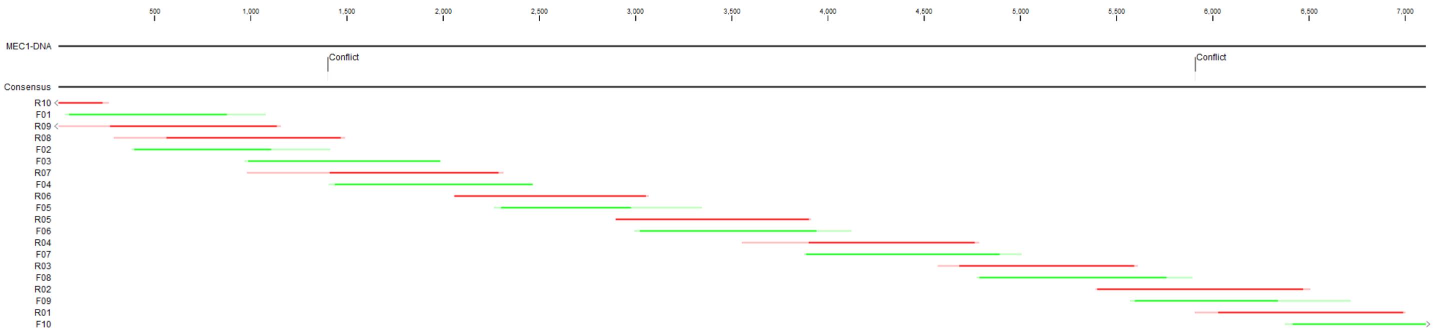

1400 G→A (C467Y)

5908 C→T (silent)

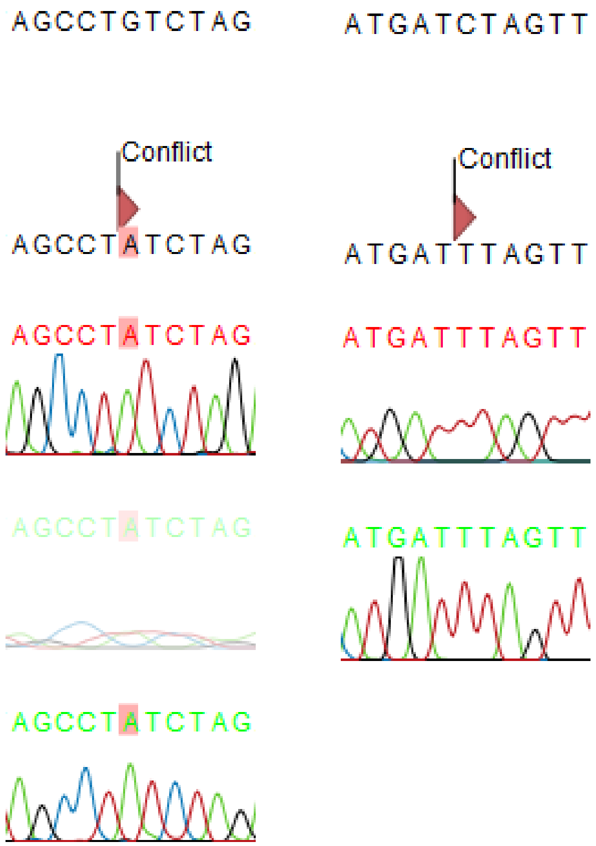

Poor coverage:

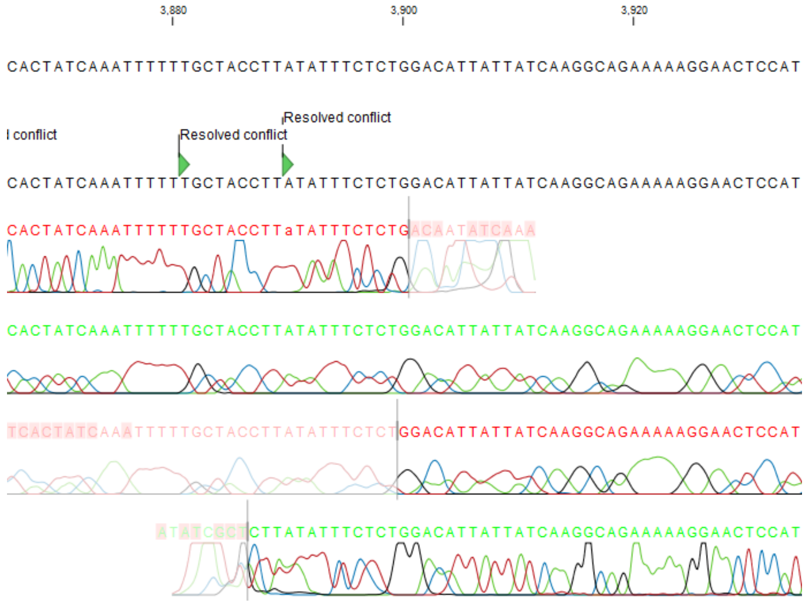

FIGURE 8 – EW10

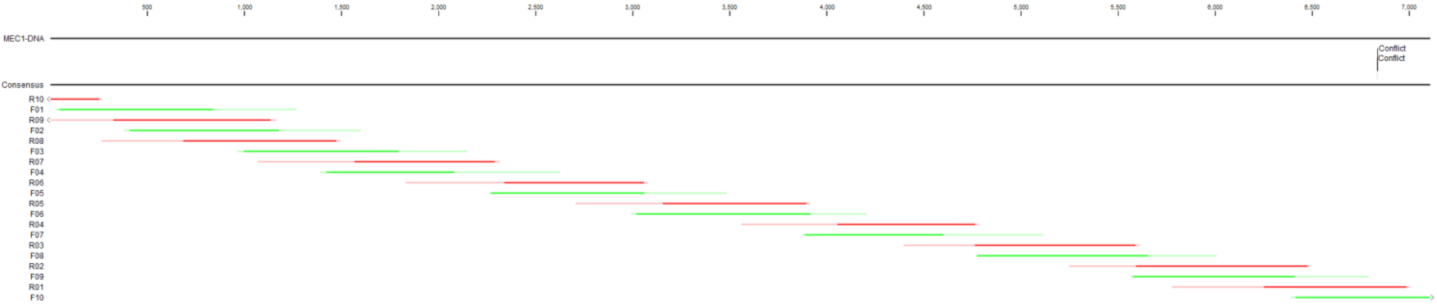

6835 G→A (G2279K)
6836 G→A (G2279K)

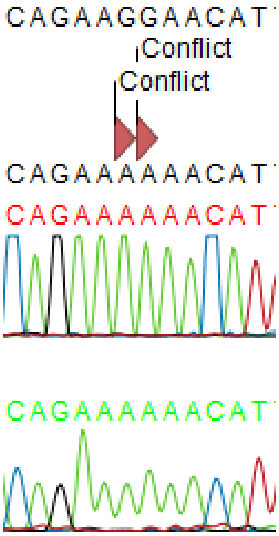

FIGURE 9 – EW 15

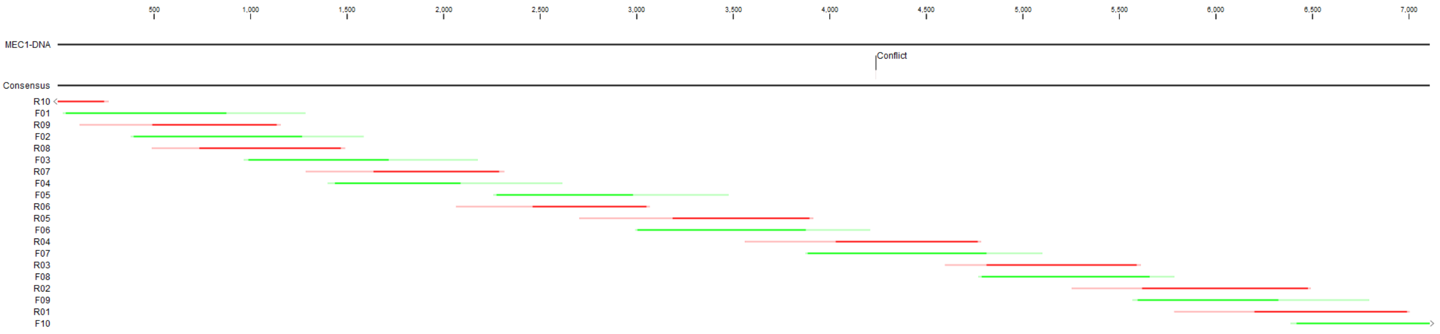

4238 C→T (S1413F)

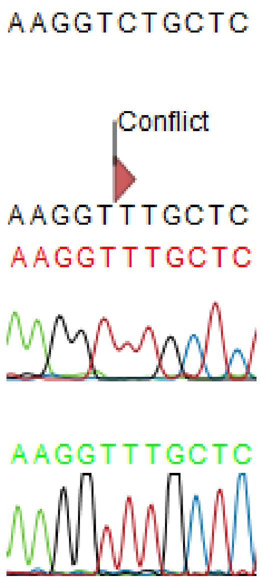

Poor coverage:

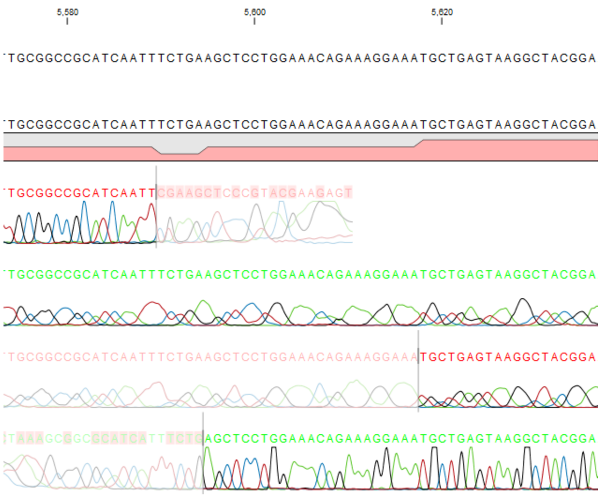

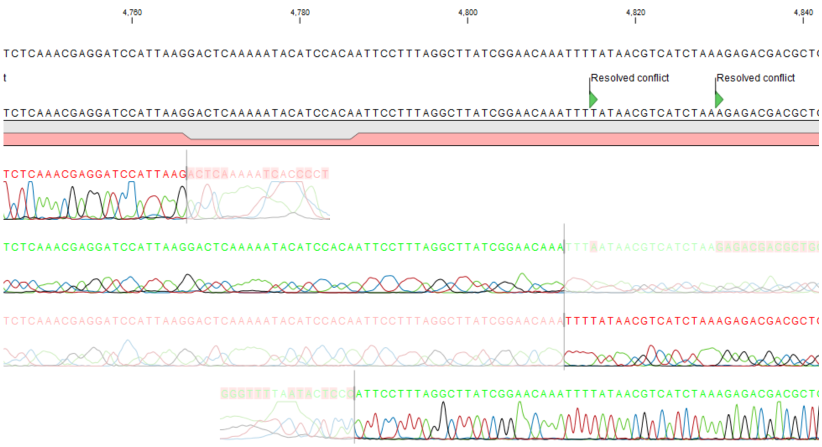

FIGURE 10 – EW 21

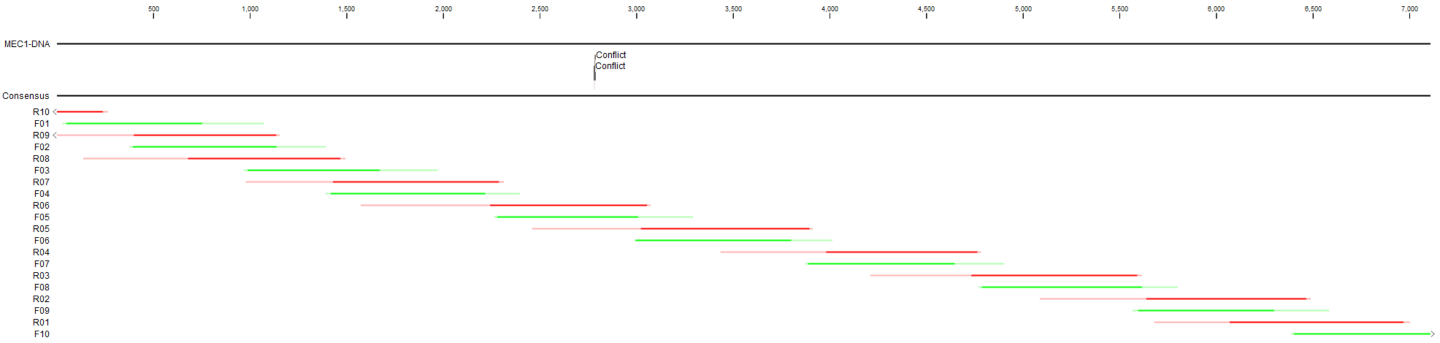

2779 G→A (A927T)
2784 G→A (silent)

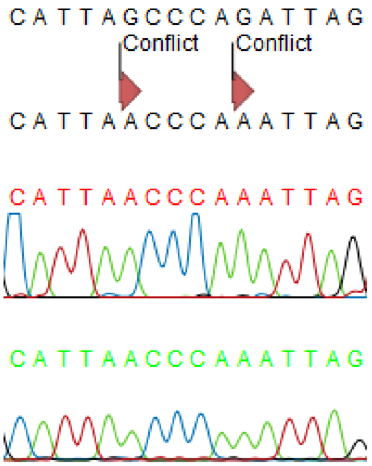

FIGURE 11 – EW 23

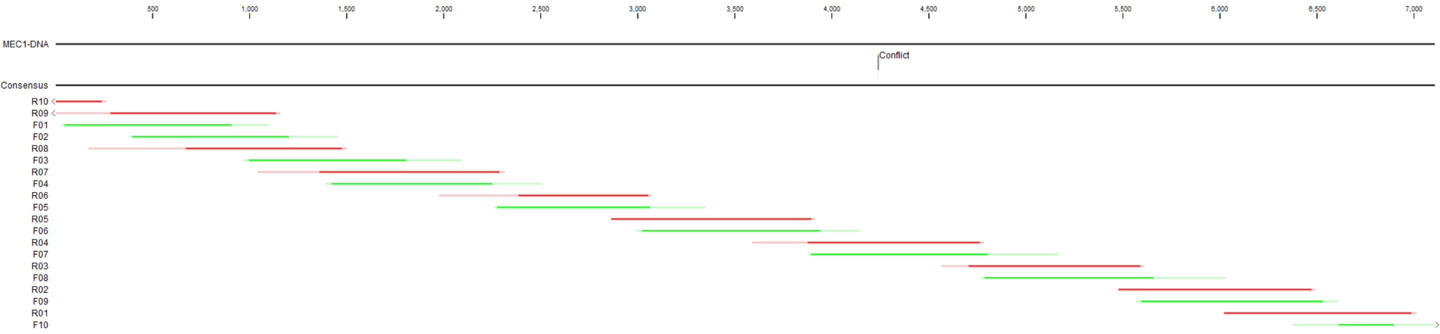

4238 C→T (S1413F)

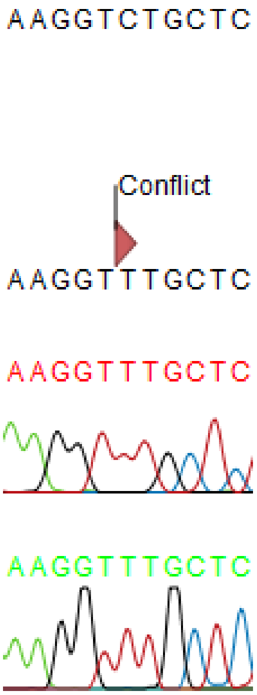

FIGURE 12 – EW 29

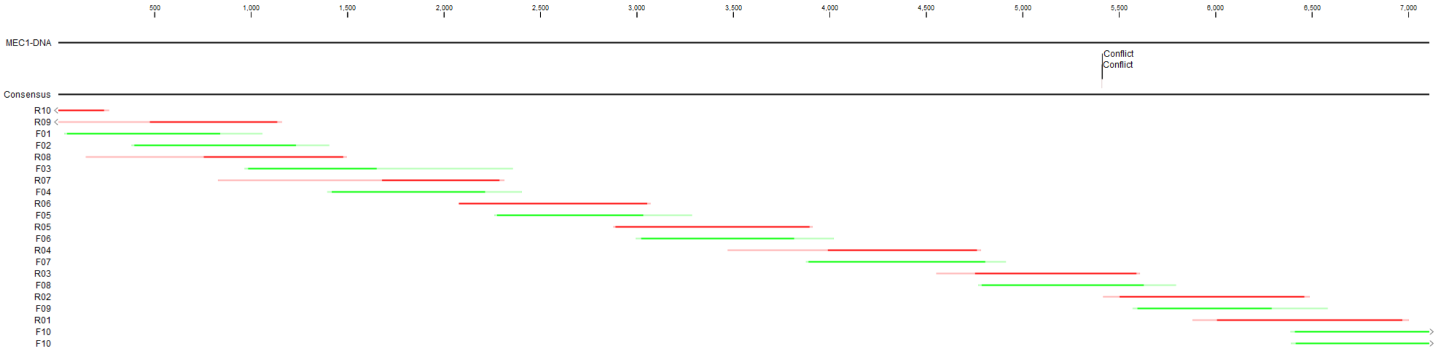

5410 G→A (G1804N)
5411 G→A (G1804N)

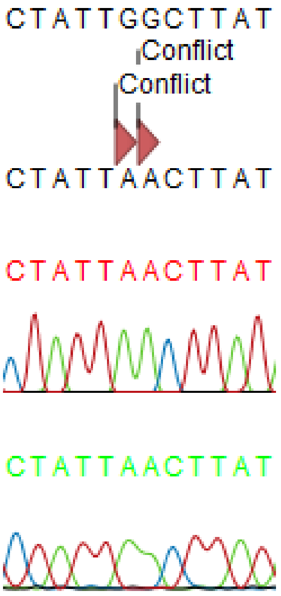

FIGURE 13 – EW 32

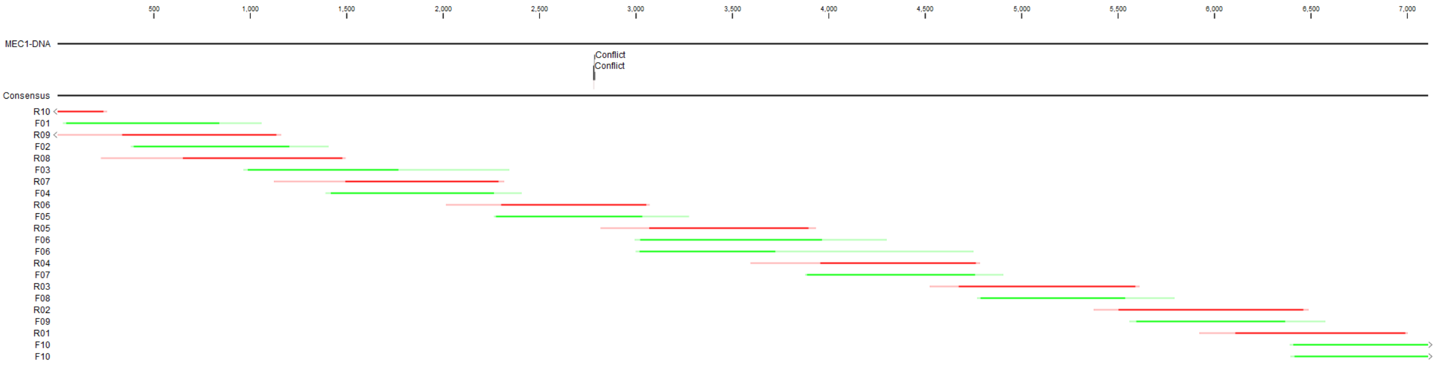

2779 G→A (A927T)
2784 G→A (silent)

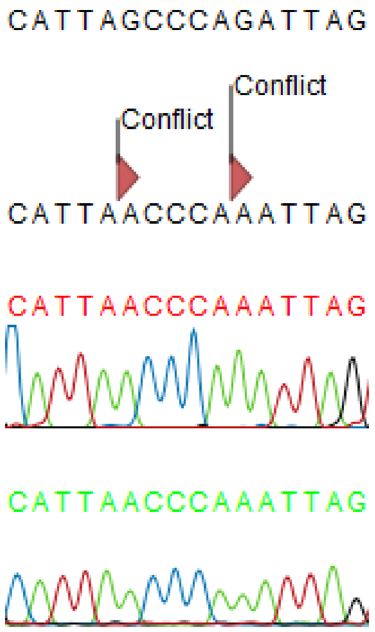

FIGURE 14 – EW 33

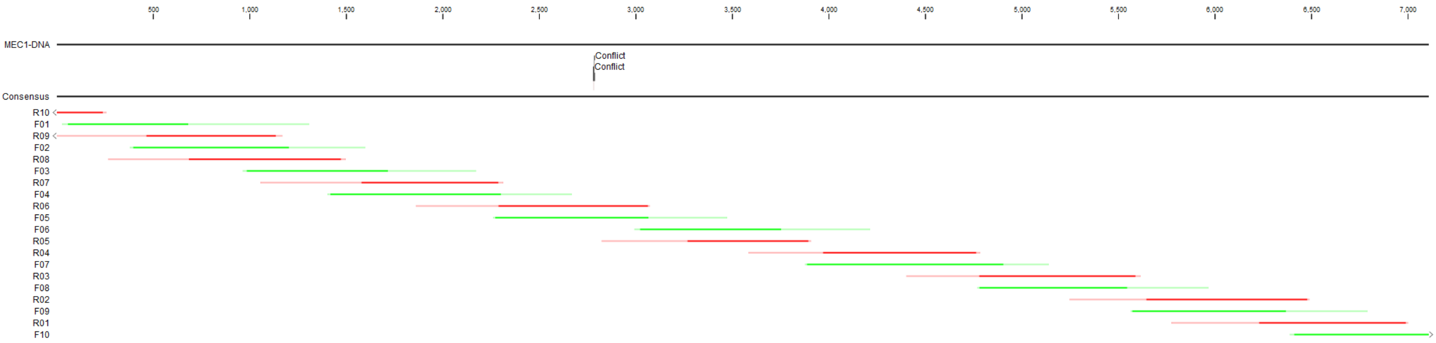

2779 G→A (A927T)
2784 G→A (silent)

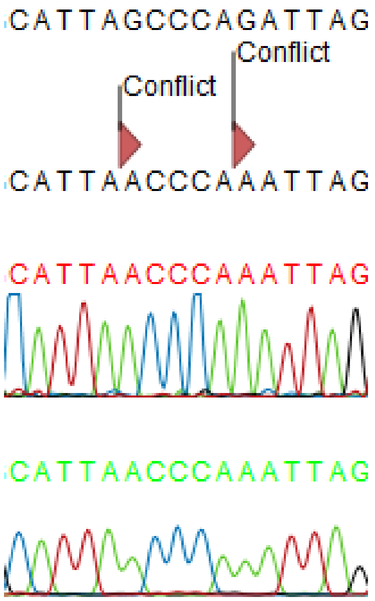

FIGURE 15 – EW 39

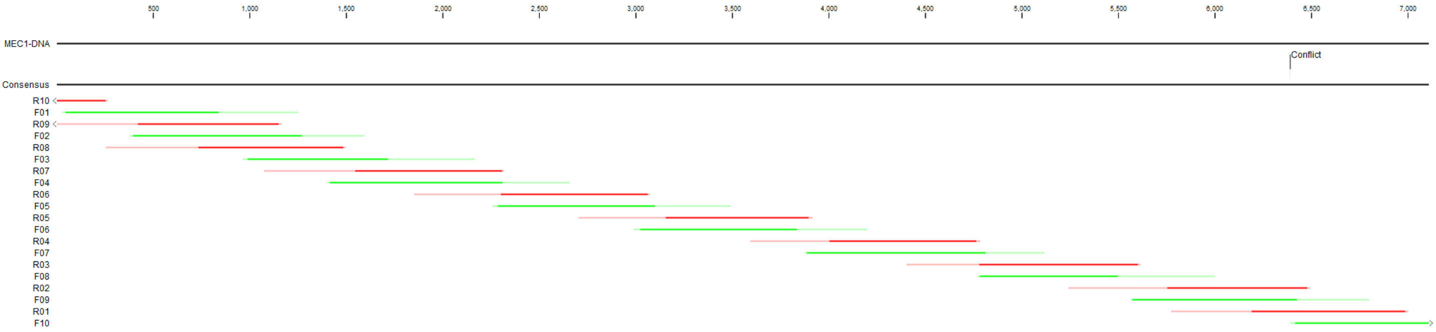

6388 G→A (E2130K)

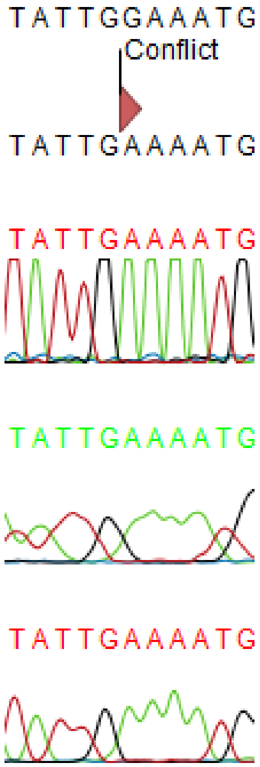

FIGURE 16 – EW 40

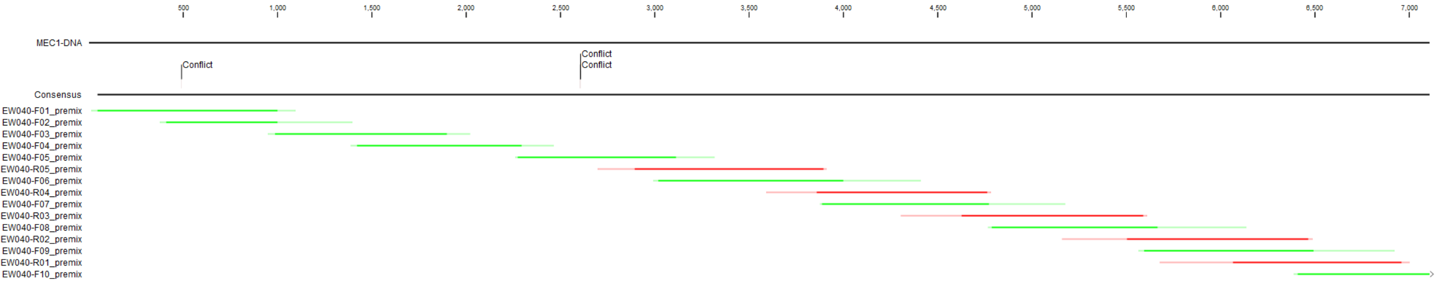

490 G→A (V164I)

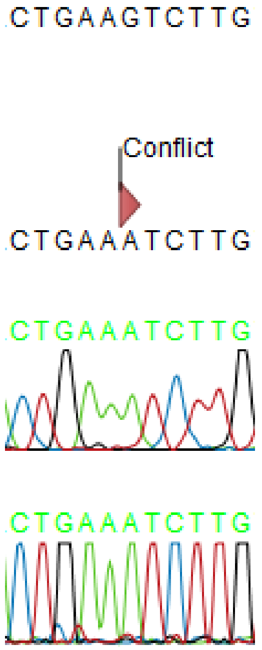

2604 G→A (Silent)
2605 G→T (E867X)

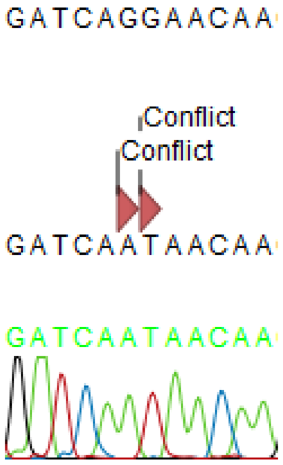

FIGURE 17 – EW 46

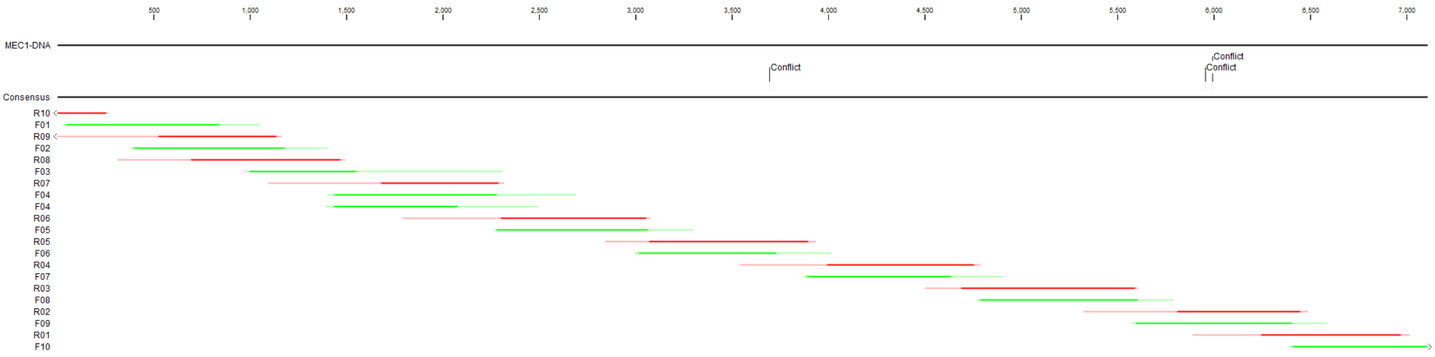

3693 G→A (silent)

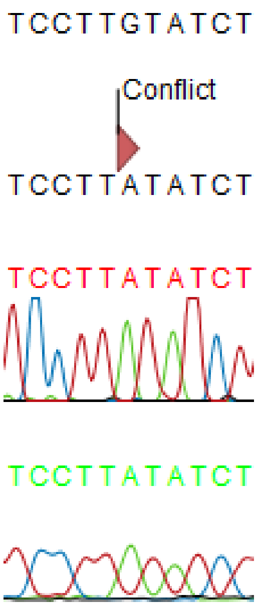

5954 G→A (C1985Y)

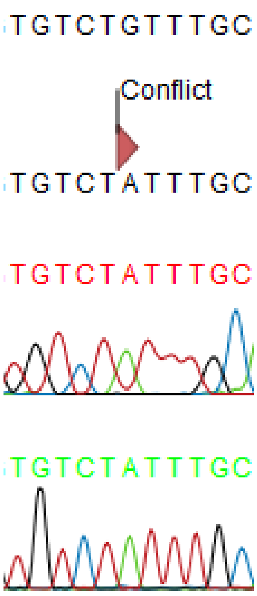

5991 C→T (silent)

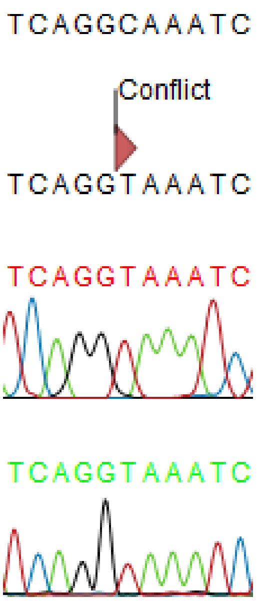

Poor coverage:

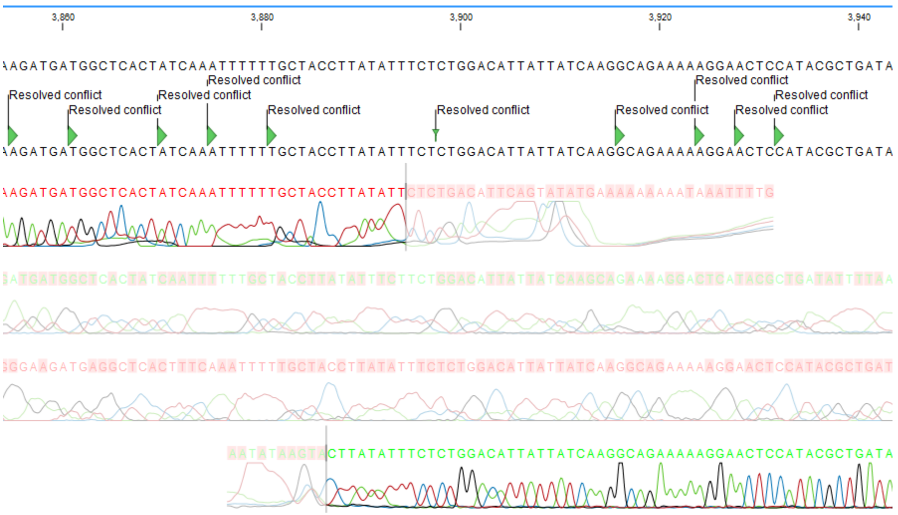

Supplement: Supplementary file 8 — Supplementary Data 5 [file 42003_2021_1884_MOESM8_ESM.pdf]
