# Supplementary material for: Cancer genome datamining and functional genetic analysis implicate mechanisms of ATM/ATR dysfunction underpinning carcinogenesis
Source: Commun Biol. 2021 Mar 19;4:363. doi: 10.1038/s42003-021-01884-x (PMC7979806; doi:10.1038/s42003-021-01884-x)
Supplement: Supplementary file 9 — Supplementary Data 6 [file 42003_2021_1884_MOESM9_ESM.pdf]

Supplementary Data 6. Clustal Omega multiple sequence alignment of Mec1, ATR, ATM, and Tel1.

FASTA sequences of Mec1, ATR, ATM, and Tel1(Fig S1A) were used as input for Clustal Omega alignment program (https://www.ebi.ac.uk/Tools/msa/clustalo/) (Supplementary Methods). The 15 Mec1 residues and the nature of mutations examined in current study are shown in red (Figure 5a).

```
MEC1      MESHVKYLDELILAIKDLNSGVDSK-----------------------------------      25
ATR       MGEHGLELASMIPALRELGSATPEEYNTVVQKPRQILCQFIDRILTDVNVVAVELVKKTD      60
ATM       ---MSLVLNDLLICCRQLEHDRATERKKEVEKFKRL-IRDPETIKHL--------DRHSD      48
TEL1      -----MEDHGIVETLNFLSSTKIKERNNALDELTTILKEDPERIPTK--------ALSTT      47
                    ::   . *      :
MEC1      ------------------------------------V--------QIKKVPTDPSSSQ--      39
ATR       SQPTSVMLLDFIQHIMKSSPLMFVNVSGSHERKGSCIEFSNWIITRLLRIAATPSCHLLH      120
ATM       SKQGKYLN----------WDAVFRFLQKYIQKETECLRIAKP------NVSASTQ-ASRQ      91
TEL1      ------------------AEALVELLASEH--TKYCDLLRNL------TVSTTNKLSLSE      81
                                                           : :  .
MEC1      --------------------EYA-KSLKILNTLIRNLKDQ--RRN--------------N      62
ATR       KKICEVICSLLFLFKSKSPAIFG-VLTKELLQLFEDLVYL--HRR--------------N      163
ATM       KKMQ-EISSLVKYFIKCANRRAPRLKCQELLNYIMDT-VKDSSNGAIYGADC-SNILLKD      148
TEL1      NRLS-TISYVLRLFVEKSCERFKVKTLKLLLAVVPELMVKDGSKSLLDAVSVHLSFALDA      140
                                     : *   . :       .
MEC1      IMKNDTIFSKTVSAL--ALLLE-YNPFLLVMKDSNG----------------------NF      97
ATR       VMGHAVEWPVVM------------SRFLSQLDEHMG----------------------YL      189
ATM       ILSVRKYWCEISQQQWLELFSVYFRLY---LKPSQDVHRVL------VARIIHAVTKGCC      199
TEL1      LIKSDPFKLKFMIHQWISLVDKICEYFQSQMKLSM-VDKTLTNFISILLNLLALDTVGIF      199
          ::                        :   :.
MEC1      EIQRL------IDDFLNIS-------------------V--------LNYDN--------      116
ATR       QSAPLQLMSMQNLEFIEVT-------------------L------LMVLTRI--------      216
ATM       SQTDG--LNSKFLDFFSKAIQCARQEKSSSGLNHILAALTIFLKTLAVNFRIRVCELGDE      257
TEL1      QVTRT--ITWTVIDFLR-------LSKKENGNTRLIMSLI--------NQLILKCHCFS-      241
          .            :*:                      :
MEC1      -------YHRIWFM----------------RRKLGSWCKACVEF-YGKPAKFQLTAHFEN      152
ATR       -------IAIVFFR----------------RQELLLWQIGCVLLEYGSPKIKSLAISFLT      253
ATM       ILPTLL---YIWTQHRLNDSLKEVIIELFQLQIYIHHPKGAKTQEKGAYES---------      305
TEL1      VIDTLMLIKEAWSYNL-----------------TI----GCTSN----------------      264
                     :                           ..
MEC1      TMNL---------------YEQALTEVLLGKTELLKFYDT-LKGLYILLYWFTSEYSTF-      195
ATR       ELFQLGGLPAQPASTFFSSFLELLKHLVEMDTDQLKLYEEPLSKLIKTLFPFE-------      306
ATM       ---------------------------TKWRSILYNLYDLLVNEIS-----HIGSRGKYS      333
TEL1      ----------------------------ELVQDQLSLFDVMSSELMNHKLPYMIGQENYV      296
                                             .:::   . :      .
MEC1      -----------------------------------GNSIAFLDS----------------      204
ATR       ------------------------------------------------------------      306
ATM       SGFRNIAVK----ENLIELMADICHQVFNEDTRSLEISQSYTTTQRESSDYSVPCKRKKI      389
TEL1      EELRSESLVSLYREYILLRLSNYKPQLFTVNH----VEFSYIRGSRD-------------      339

MEC1      SLGF---------AKFDFNFQRLIRIV----------LYVFDSCELAPLEYAEIQLKYIS      245
ATR       -----------------------------------------------AEAYRNIEPVYLN      319
ATM       ELGWEVIKDHLQKSQNDFDLVPWLQIAT---QLISKYPASLPNCELSPLL-----MILSQ      441
TEL1      KNSWFALPDFRLRDR--GGRSVWLKILGITKSLLTYFALNRKNENY------------SL      385

MEC1      LVVDYVCNRTISTA----------------------LDAPALVCCEQLKFVLTTMHHFLD      283
ATR       MLLEKLCVMFEDGV----------LMR-------LKSDLLKAALCHLLQYFLK----FVP      358
ATM       LLPQQRHGERTPYVLRCLTE---------------------VALCQ----------DKRS      470
TEL1      LFKRRKCDSDIPSILRISDDMDTFLIHLLEENSSHEFEVLGLQLCSFYGTLQ----DFTK      441
          :.                                          *
MEC1      NKYGLLDNDPTMAKGILRLYSLCISNDFSKCFVDH--FPIDQWA-----DFSQSEHFPFT      336
ATR       AGYES-------ALQVRKVYVR----NICKALLDV--LGIEVDA-----EYLLGPLYAAL      400
ATM       NLESSQK------SDLLKLWNK----IWCITFRGISSEQIQAENFGLLGAIIQGSLVEVD      520
TEL1      SFAEQLK------ELLFSKFEK----IQCFNWVCFSFIPLLSQKEC---ELSNGDMA---      485
                         :   :        .          :             .
MEC1      Q----------------------LT---------NKALSIVYFDLKRR---SL-------      355
ATR       KMESMEIIE----EIQCQTQQENLSSNSDGISPKRRRLSSSLNPSKRAPKQTE-------      449
ATM       R-EFWKLFTGSACRPSCPAVCCL-----------TLALTTSIVPGTVKMGIEQNMCEVN-      567
TEL1      R-LFKVCLPLVKSNESCQLSCL------------LLANSIKF--SKQLLSDEKTINQIYD      530
          :                                     :      .
MEC1      -------PVEALKYDNKFNIWVYQSEPDSSLK------NVTSPFDDRYKQLE---KLRLL      399
ATR       -------EIKHVDMNQKSILWSALKQKAESLQISLEYSGLKNPVIEMLEGIAVVLQLTAL      502
ATM       ---------RSFSLKESIMKWLLFYQLEGDLENSTEVP------PILHSNFP---HL-VL      608
TEL1      LYELSDILGPILVTNESFMLWGYLQYVGKDFQSMNGISSADRIFEWLKSKWN---QL-RG      586
                     .  .:.   *        .::                .      :*
```

```
MEC1        V----------LKKF---------NKTERGTL--L---KYRVNQLS-PGFFQRAGNDFKL      434
ATR         CTVHCSHQNMNCRTF---------KDCQHKSKKKP---SVVITWMS-LDFYTKVLKSCRS      549
ATM         EKILVSLTMKNCKAAMNFFQS--VPECEHHQK-----DKEELSFSEVEELFLQTTFDKMD      661
TEL1        TDA---KQDQFCN-FISWLGNKYDPENPFNDKKGEGANPVSLCWDESHKIWQHF-QEQRE      641
                        .            .               :   .   :: :   .
MEC1        ILN---------------------------------------------------------      437
ATR         LLESVQKLDLE-------------------------------------------ATIDK      565
ATM         FLTIVRECGIEKHQSSIGFSVHQNLKESLDRCLLGLSEQLLNNYSSEITNSETLVRCSRL      721
TEL1        FLLG-----VKPEEKSECF----------NTPFFNLPKV-----------SLDLTRYNEI      675
            :*
MEC1        -----EA--SVSI---QTCFK------------TNNIT-----RLTSWTVILGRLACLES      470   C467Y
ATR         VVKIYDALIYMQV---NSSFE------------DHILEDLCGMLSLPWIYSHSDDGCLKL      610
ATM         LVGVLGCYCYMGVIAEEEAYKSEL--FQKAKSLM-----QCAGESITLFKNKTNE-EFRI      773
TEL1        LYRLLE------N-IESDAFSSPLQKFTWVAKLIQIVDNLCGDSTFSEFIAA-----YKR      723
                            . .:.                                     .
MEC1        EKFSGTLPNST---------------------------------KDMDNWYVCHLCDIEK      497
ATR         TTFAANLLTLS--CRISDSYSPQAQSRCVFLLTLFPRRIFLEWRTAVYNWALQSSHEVIR      668
ATM         GS-LRNMMQLCTRCLSNCTKKSPNKIASGFFLRLLTSKLMN---------------DIAD      817
TEL1        TT-LITIPQLS--------FDSQ-NSYQSFFEEVLSIRTIN-----VDHL----VLDKIN      764
             .   .:                                                 :
MEC1        T---GNPFVRINPNRPEAAGKSEVFRILHSNFLSHPNI-DEFNES---------LLSGIL      544
ATR         ASCVSGFFILLQQQN----SCNRVPKILIDKVKDDSDIVKKEFAS---------ILGQLV      715
ATM         ICKSLASFIKKPFDRGEVESMEDDTNGNLMEVEDQSSM-NLFNDYPDSSVSDANEPGESQ      876
TEL1        MKEIVNDFIRMQKNKSQ---------------TGTSAI-NYFEASSE----DTT-QNNSP      803
                   *:    :.                  .   : .                .
MEC1        FSLHRIFSH---FQPPKLTDGNGQI-NKSFKL-----VQKCFMN------SNRYLRLLST      589
ATR         CTLHGMFYLTSSLTEPFSEHGHVDLFCRNLKATS---QHECSSSQLKASVCKPFLFLLKK      772
ATM         STIGAI----------------------------NPLAEEYLSK----------------      892
TEL1        YTIGGR-------------------FQKPLHSTIDKAVRAYLWSSRNKSISERLVAILE-      843
             ::                                   .    .
MEC1        RIIPLFN---IS---------DSHNSEDE-------------------------------      606
ATR         KIPSPVKLAFIDNLHHLCKHLDFREDETD-------------------------------      801
ATM         -----QDLLFLDMLKFLCLCVTTAQTNTVSFRAADIRRKLLMLIDSSTLEPTKSLHLHMY      947
TEL1        FSDCVSTDVFISYLGTVCQWLKQAIGEKSSY-----------------------------      874
                      :.              :
MEC1        ------------------HT---ATLIKFLQ--SQKL-----------------------      620
ATR         ------------------VKAVLGTLLNLME--DPDKDVRVAFSGNIKHILESLD-----      836
ATM         LMLLKELPGEEYPLPMEDVLELLKPLSNVCSLYRRDQDVCKTILNHVLHVVKNLGQSNMD      1007
TEL1        ------------NKILEEFTEVLG-EKLLCNHYSSSNQA---------------------      900
                                        . .    .
MEC1        ---------PV---V----------KEN----LVIAWTQLTLTTSNDVFDTLLLKLIDIF      654
ATR         SE------DGF---I----------KELFVLRMKEAYTHAQISRNNELKDTLILTTGDIG      877
ATM         SENTRDAQGQFLTVIGAFWHLTKERKYIFSVRMALVNCLKTLLEADPYSKWAILNV----      1063
TEL1        ----MLLLTSYIEAIRPQWLSYPEQPL-------NS-------DCNDILDWIISRF----      938
                          :                              :   .  :
MEC1        NSDDYSLRIMMTLQIKNMAKILK-----KTPYQLLSPILPVLLRQLGKNL------VERK      703
ATR         RAAK-----------------GD-----LVPFALLHLLH----CLLSKSA------SVSG      905
ATM         -------------MGKDFPVN-------EVFTQFLADNHHQ-VRMLAAESINRLFQDTKG      1102
TEL1        -------------EDNSFTGVAPTVNLSMLLLSLL-QNHDL-----S-------HGSIRG      972
                                             :*           .
MEC1        ---------------VGFQNLIELLGYSSKTIL--DIFQRYIIP--------------Y-    731
ATR         ---------------AAYTEIRALVAAKSVKLQ--SFFSQYKKP--------------I-      933
ATM         DSSRLLKALP-------------------LKLQQTAFENAYLKAQEGMREMSHSAENPE-      1142
TEL1        GKQRVFATFIKCLQKLDSSNIINIMNSISSYMAQVSYKNQSIIF----YEIKSLFGPPQQ      1028
                                           :      .
MEC1        --A---IIQYKSDVLSEIAKI-----MCDGD-------TSL------INQMKVNLLKKNS      768
ATR         --CQFLVESLHSSQMTALPNT-----PCQNA-------DVRKQDVAHQREMALNTLSEI-      978
ATM         --TLDEIYNRKSVLLTLIAVVLSCSPICEKQALFALCKSVKENGL--EPHLVKKVLEKVS      1198
TEL1        SIEKSAFYSLAMSMLSLVS---------YPSLVFSLEDMMTYSGFNHTRAFIQQALNKIT      1079
                  . .     :: :                                :  : *.:
MEC1        RQIFAVALVKHGLFSLDILETLF-----------L---------------------NRAP      796   A775P
ATR         -----------ANVFDFPDLN---------------------------------------      988
ATM         -----------ETFGYRRLEDFMASHLDYLVLEWLNLQDTEYN--LSSFPFILLNYTNIE      1245
TEL1        -----------VAFRYQNLTELFEYCKFDLIMYWFNRTKVPTSKLEKEWDISLFGFADIH      1128
                         *    *
MEC1        TFDKGYITAYLPDYKTLAEITKL-YKNSVTKDASDSENANMILCSLRFLITNF-------      848
ATR         RFLTRTLQVLLPDLAAKASPAASALIRTLGKQ-LNVNRREILINNFKYIFSHL----VCS      1043
ATM         DFYRSCYKVLIPHLVIRSHFDEVK---SIANQ-IQEDWKSLLTDCFPKILVNILPYFAYE      1301
TEL1        EFLGRYFVE-ISAIYFSQG--------------FNQKWILDMLH-------------AIT      1160
             *        :                       : .    :
MEC1        -EKD-------------------------------------------KRHGSKYKNI---      861
ATR         CSKDEL--------ERALHYLKNETE--IELGSLLRQDFQGLHNELLLRIGEHYQQVFN-      1092
ATM         GTRDSGMAQQRETATKVYDMLKSENLLGKQIDHLFISNLPEIVVELLMTLHEPAN-----      1356
TEL1        GNGDAYLV------DNSYYLCIPLAFISGGVNELIFDILPQISGKTTVKYHKKYRLLMLK      1214
               *                                               .  .
```

```
MEC1      --------------NSWT--DD---------------QEQAFQKKLQDNILG------IF      884
ATR       ---------GLSILASFASSDD--PYQGP----RDIISPELMADYLQPKLLG------IL      1131
ATM       -----SSASQSTDLCDFSGDLDPAP--NPPHFPSHVIKATFAYISNCHK----TKLKSIL      1405
TEL1      WIIRFTDLGSLTELRSTVEKLFPTSYLSPYLFENSSVSMRYQYPLHIPLALGATLVQTQF      1274
                         .                     .                     :
MEC1      Q-----------VFSSDIHDVEGRTTYYEKLRVING-ISFLIIYAP--------------      918
ATR       A-----------FFNMQLLSSSV--GIEDKKMALNSLMSLMKLMGP--------------      1164
ATM       EILSKSPDSYQKILLAICEQAAETNNVYKKHR----ILKIYHLFVSLLLKDIKSGLGGAW      1461
TEL1      AHEKNNTHEFKLLFLSVITDLEKTSTYIGKLRCARELKYLFVLYENVLVKSS------TL      1328
                      .:     .         *         :  :
MEC1      --------KKSIISALAQISICL---------QTGLGLKEV-RYEAFRCWHLLVRHLNDE      960     A929T
ATR       --------KH-VSSVRVKMMTTL---------RTGLRFKDDFPELCCRAWDCFVRCLDHA      1206
ATM       AFVLRDVIYTLI-HYINQRPSCI----MDV-SLRSFSLCCDLLSQVCQTAVTYCKDALEN      1515
TEL1      NFIIIRLSKFLIDTQIHDEVITIFSSLLNLADKNTFEIEPSLPNLFCKIFI-YLRENK--      1385
                     :     .    :            : :         :      :
MEC1      ELSTVIDSLIAFILQKWSEFNGKLRNIVYSILDTL---IKEKSDLILKLKPYTTLA----      1013
ATR       CLGSLLSHVIVALLPLIH-IQPKETA---AIFHYL---IIENRDAVQDFLH-EIYF----      1254
ATM       HLHVIVGTLIPLVYE-----QVEVQKQVLDLLKYL---VIDNKDN---ENLYITIKLLDP      1564
TEL1      QLSPSFQQAIKLLEH-----RDLIK---IKTWKYFLDAIFGNIVQ---DDIYENTELLDA      1434
           *   .   *  :       .           . :   :  :
MEC1      LVGKPELGILARDGQFARMVNKIRSTTD-LIPIFANNLKSS---NKYVINQNLDDIEVYL      1069
ATR       LPDHPELKKIKAV--LQEYRKETSESTD-LQTTLQLSMKAIQHENVDVRIHALTSLKETL      1311
ATM       FPDHVVFKDLRITQQKIKYSRGPFSLLEEINHFLSVSVYDA---LPLTRLEGLKDLRRQL      1621
TEL1      SDC--GVDDVVLVSLLFSYARRPVAS----------------------KIGC-------      1462
                .  :          .
MEC1      R-RKQTERSIDFTPKKVGQTSDITLVLGALLDTSHK--FRNLDKDLCEKCAKCISMIGVL      1126     G1124N
ATR       Y--KNQEKLIKYATDSETVEPIISQLVTVLLKGCQD-----ANSQARLLCGECLGELGAI      1364
ATM       ELHKDQMVDIMRASQDNPQDGIMVKLVVNLLQLSKMAINHTGEKEVLEAVGSCLGEVGPI      1681
TEL1      SLSKAAAINILKH--HVPKEYLSKNFKLWFAALSRRILQQEVQRER---STNFNNEVHLK      1517
             *     *               .   :   .:       : :      .  . :
MEC1      DVTKHEFKRTTYS-----ENEVYDLNDSVQTIKFLIWVINDILVPAFWQSENPSKQLFVA      1181
ATR       DPGRLDFSTTETQ-----GKD-FTFVTGVEDSSFAYGLLMELTRAYLAYADNSRAQDSAA      1418
ATM       DFSTIAIQHS--------KDASYTKALKLFEDKELQWT-FI-MLT---YLNNT-------      1721
TEL1      NFEMV-FRHPEQPHMIYQRISTFNKEAELYDSTEVFFISEC-ILT---YLVGY-------      1565
          :     :               :     :   .                  .
MEC1      LVIQESLKYCGLSSESWDMNHKELYPNEAKLWEKFNSV---SKTTIYPLLSSLYLA----      1234
ATR       YAIQELLS-------IYDCREMETNGPGHQLWRRFPEH---VREILEPHLNTRYKSSQ--      1466
ATM       -LVEDCVKV--RSAAVTCLKNILATKTGHSFWEIYKMTTDPMLAYLQPFRTSRKKFLEVP      1778
TEL1      -SIG-------NSESEFCFRDNIMNENKDKVAPLD---K-DVLNAIYPL-----------      1602
            :                ..        ..              : *
MEC1      -----QSWKEYVP-LKYPSNNFKEGYKIWVKRFTLDLLKTGT-TENHPLHVFSSLIREDD      1287
ATR       ---KSTDWSGVKK-PIYL-SKLGSNFAEWSASWAGYLITKVRHDLASKIFTCCSIMMKHD      1521
ATM       RFDKENPFEGLD--DINLWIPLSENHDIWIKTLTCAFLDSGGT--KCEILQLLKPMCEVK      1834
TEL1      ----ANNF-GMESFICDTYLSVNEPYNCWLSKFARSLIHQISF--NIPPIVCLYPLCKGS      1655
                 :             . . .  *    :  ::                 : : .
MEC1      GSLSNFLLPYISLDIIIKAEK---GTPYADILNGIIIEFDSIFTCNLEG---------MN     1335
ATR       FKVTIYLLPHILVYVLLGCNQEDQQEVYAEIMAVLK--HDDQHTIN------------TQ     1567
ATM       TDFCQTVLPYLIHDIL--LQD--TNESWRNLLSTHV---QGFFTSCLRHFSQTSRSTTPA     1887
TEL1      TAFCELVLTDLFFLSTTYDPK--SCLNWSNRIFTQI---AMLLHV-----KD--------     1697
            .   :*  :         .      : : :
MEC1      NLQVDS--------LRMCYESIFRVFEYCKKWATEFKQNYSKLHGTFII---------KD      1378
ATR       DIASDL--------CQLSTQTVFSMLDHLTQWARHKFQALKAEKCPHSKSNRNKVDSMVS      1619
ATM       NLDSESEHFFRCCLDKKSQRTMLAVVDYMRRQKRPSSGTIF-------------------      1928
TEL1      ---SEI-----------KLKMLFNVIKMIRMGSRCKERNCL-------------------      1724
              :              . :: :..
MEC1      TKTTNMLLRIDEFLRTTPSDLLAQRSLETDSFERSALYLEQCYRQNPHD-----------      1427     S1413F
ATR       TVDYEDYQSVTRFLDLIPQDTLAVASFRSKAYTRAVMHFESFITEKKQN-----------      1668
ATM       ---------NDAFWLDLNYLEVAKVAQS-CAAHFTALLYAEIYADKKSMDDQEKRSLAFE      1978
TEL1      -----------RIYSSLDLQEICQISLKIKEFKFGYLLFEEMNM-----PN--IRE----      1762
                      :        :.  :          :   .
MEC1      ----------------KNQNGQLLKNLQITYEEIGDIDSLDGVLRTFAT-GNLVSKIEEL      1470
ATR       ----------------IQE---HLGFLQKLYAAMHEPDGVAGVSAIRKAEPSLKEQILEH      1709
ATM       EGSQSTTISSLSEKSKEETGISLQDLLLEIYRSIGEPDSLYGCGGGKMLQPITRLRTYEH      2038
TEL1      ------------------MN---INTLQKIYECINDGDFLAGLPVPHSIEGVLNSINRID      1801
                                    *   *  : : * : *
MEC1      QYSENWKLAQDC---FNVLGKFSEDPKT-TTRMLKSMYDHQLYSQIISNSSFHSSDGEIS      1526
ATR       ESLGLLRDATAC---YDRAIQLEPDQIIHYHGVVKSMLGLGQLSTVI-------------      1753
ATM       E--AMWGKALVT---YDLETAIP--SSTRQAGIIQALQNLGLCHI---------------      2076
TEL1      S--DTWKRFLFNNADFDANYTTS--LEEEKESLIKATEDSGFYGL---------------      1842
          .              ::               ::::  .
MEC1      LSPDVKEWYSIGLEAANLEGNVQTLKNWVEQIESLRNIDDREVLLQYNIAKALIAISNED      1586     G1546S
ATR       ---------------TQVNGVHANRSEWTDELNTYRVEAA-WKLSQWDLVENYLAADGKS      1797
ATM       -------------LSVYLKGLDYENKDWCPELEELHYQAA-WRNMQWDHCTSVSKEVEG-      2121
TEL1      ----------TSLLESRLSGSS-DVYKWNLELG------------DWKLLTPKVVD----      1875
                           :.*      .*  ::            ::.
MEC1      PL---------------RTQKYIHNSFRLIGTNFITSS----------KETTLLKKQNLL      1621
ATR       TTWSVRLG-QLLLSAKKRDITAFYDSLKLVRAEQIVPLSAASF-----ERGSYQRGYEYI      1851
```

```
ATM       TSYHESLYNALQSLRD-REFSTFYESLKYARVKEVEEMCKRSLESVYSL-------YPTL      2173
TEL1      -SKAKGLYYAIKNLPQDVGFA--------------EKSLEKSLLTIFDSRQHFISQTEWM      1920
                                                                     :
MEC1      MKLHSLYDLS------FLSSAKDKFEYKSNTTILDYRMER-IGADFVPNHYILSMRKSFD      1674
ATR       VRLHMLCELEHSIKPLFQHSPGDSSQEDS--LNWVARLEM-TQNSYRAKEPILALRRALL      1908
ATM       SRLQAIGELE-SIGELFSRSVTHRQLSEVYIK-WQKHSQLLKDSDFSFQEPIMALRTVIL      2231
TEL1      DTLNAIIEFI-KIAAIPQD-VTS--FPQTLMSIMKADKERLNTIDFYDHKTTLKSRHTLM      1976
            *: : ::                  .          :     .:  :.  :  *  :
MEC1      QLKMNEQADADLGKTFFTLA-----------QLARNNARL-----------------DIA      1706
ATR       SLNKRPDYNEMVGECWLQSA-----------RVARKAGHH-----------------QTA      1940
ATM       EILMEKEMDNSQRECIKDILTKHLVEL--------------------SILARTFKNTQL-      2270
TEL1      NVLSRNSLDE-NVKCSKYLRLGSIIQLANYVQLAIANGAPQDALRNATLMSKTVKNIAKL      2035
          .:  . . :    :
MEC1      S--------ESLMHCLERRLPQAELEFAEILWKQGENDRALKIVQEIHEKY-QENSSV--      1755      A1726V
ATR       Y--------NALLNAGESRLAELYVERAKWLWSKGDVHQALIVLQKGVELCFPENETPPE      1992
ATM       --PERAIFQIKQYNSVSCGVSEWQLEEAQVFWAKKEQSLALSILKQMIKKLDASCA----      2324
TEL1      YDDPSVVSQIEKLAS---------FTSANALWESREYKAPVMIMRDLLAQNEKNISESIL      2086
                        .         .  *: :* . :    : :::.       .
MEC1      -------NARDRAAVLLKFTEWLDLSNNSASEQIIKQYQDIF-QIDSKWD----------      1797
ATR       GK-----NMLIHGRAMLLVGRFMEETANFESNAIMKKYKDVT-ACLPEWE----------      2036
ATM       ANNPS--LKLTYTECLRVCGNWLAETCLENPAVIMQTYLEKAVEVAGNYDGESSDELRNG      2382
TEL1      YDDFKLLINVPMDQIKARLVKWSSESRLEPAAAIYEKIIV-------NWDINVED--HES      2137
                              .:   :       * :           :::
MEC1      --KPYYSIGLYYSRLLERKKAEGYITN---------GRFEYRA-----------------      1829      G1804N
ATR       --DGHFYLAKYYDKLMPMVTDNKMEKQ---------GDL---------------------      2064
ATM       KMKAFLSLARFSDTQYQRIENY-----------MKSSEFENKQALLKRAKEEVGLLREHK      2431
TEL1      CSDVFYTLGSFLDEQAQKLRSNGEIEDREHRSYTGKSTLKALELIYKNTKLPENERKDAK      2197
            . .  :. : .                       . :
MEC1      I---SYF---LLAF-------------------------E----KNTAKVRENLPKVITF      1854
ATR       I---RYI---VLHF-------------------------GRSLQYGNQFIYQSMPRMLTL      2093
ATM       IQTNRYTVKVQRELELDELALRALKEDRKRFLCKAVENYINCLLSGEE-HDMWVFRLCSL      2490
TEL1      RHYNR----VLLQYNRDSEVLKALLLQKEKFLWHALHFYLNTLVFSNRYDNDIIDKFCGL      2253
                                                       .       : :.  :
MEC1      WLDIAAASISEAPGNRKEML--SKATEDICSHVEEALQHCPTYIWYFVLTQLLSRLLHS-      1911      A1859V
ATR       WLDYGTKAYEWEKAGRSDRVQMRNDLGKINKVITEHTNYLAPYQFLTAFSQLISRICHS-      2152
ATM       WLE-------------------NSGVSEVNGMMKRDGMKIPTYKFLPLMYQLAARMGTKM      2531
TEL1      WFE-------------------NDDNSKINQLLYKEIGTIPSWKFLPWVNQIASKISME-      2293
          *::                    .   .:   : :       :  :   .  *: :::  .
MEC1      --HQSSAQIIMHILLSLAVEYPSHILWYITALVNSNSSKRVLRGKHIL-----------E      1958
ATR       --HDEVFVVLMEIIAKVFLAYPQQAMWMMTAVSKSSYPMRVNRCKEIL-----------N      2199
ATM       MGGLGFHEVLNNLISRISMDHPHHTLFIILALA------NANRDEFLTKPEVARRSRITK      2585
TEL1      --ENEFQKPLQLTMKRLLYKLPYDSLYSVMSILL--YEKQSNKDTNIS-----------Q      2338
                   :   :  :    * . :: : ::       .  :   :            :
MEC1      KYRQHSQNP------------------------HDLVSSALDLTKALTRVCLQDVKS-IT      1993      C1985Y
ATR       KAIHMKKSL-------------------------EKFVGDATRLTDKLLELCNKPVDG-SS      2234
ATM       NVPKQSSQLDEDRTEAANRIICTIRS-----RRPQMVRSVEALCDAYIILANLDATQWKT      2640
TEL1      KI------------QAVKKILLELQGYDRGAFAKKYLLPVQEFCEMSVELANLKFVQNTK      2386
          :                                 . :  .  :  .    :.        .
MEC1      SRSGKSLEKDFKFDMNVA----PSAMVVPVRKNLDIISPL--ES-NSMRGYQPFRP-VVS      2045
ATR       STL--SMSTHFKMLKKLVEEATFSEILIPLQSVMIPTLPSILGTHANHASHEPFPGHWAY      2292
ATM       QRKGINIPAD-----------QPITKLKNLEDVVVPTMEIKVDHT-------GEYGNLVT      2682
TEL1      TLRLANLKIG-----------QYWLKQLNMEKLPLPTSNFTVKSSAD-----GRKARPYI      2430
               .:                      :..
MEC1      IIRFGSSYKVFSSLKKPKQLNIIGSDGNIYGIMCKK--EDVRQDNQYMQFATTMDFLLSK      2103
ATR       IAGFDDMVEILASLQKPKKISLKGSDGKFYIMMCKP-KDDLRKDCRLMEFNSLINKCLRK      2351
ATM       IQSFKAEFRLAGGVNLPKIIDCVGSDGKERRQLVKG-RDDLRQDAVMQQVFQMCNTLLQR      2741
TEL1      VSVNETVGITTTGLSLPKIVTFNISDGTTQKALMKGSNDDLRQDAIMEQVFQQVNKVLQN      2490
          :           .:. ** :    ***.    : *   :*:*:*    :.     :  * .
MEC1      DIASRKRSLGINIYSVLSLREDCGILEMVPNVVTLRSILSTKYESLKIKYSLKS-----L      2158      G2127E
ATR       DAESRRRELHIRTYAVIPLNDECGIIEWVNNTAGLRPILTKLYKEKGVYMTGKE-----L      2406.     E2130K
ATM       NTETRRKRKLTICTYKVVPLSQRSGVLEWCTGTVPIGEFLVNNEDGAHKRYRPNDFSAFQC      2801
TEL1      DKVLRNLDLGIRTYKVVPLGPKAGIIEFVANSTSLHQILSKLHTND-------KITFDQA      2543
          :   *. .* *  *  *: *  *   .*::*   . . :  :*       .
MEC1      HDRWQ-H--TAADGKLE-FYMEQVDKFPPILYQWFLENFPDPINWFNARNTYARSYAVMA      2214
ATR       RQCML-PKSAALSEKLKVFREFLLPRHPPIFHEWFLRTFPDPTSWYSSRSAYCRSTAVMS      2465
ATM       QKKMMEVQKKSFEEKYE-VFMDVCQNFQPVFRYFCMEKFLDPAIWFEKRLAYTRSVATSS      2860
TEL1      RKGMKAVQTKSNEERLK-AYLKITNEIKPQLRNFFFDSFPDPLDWFEAKKTYTKGVAASS      2602
          :.        :  . : :        .  *  :  :  : .* **  *:.  :  :* :. *. :
MEC1      MVGHILGLGDRHCENILLDIQTGKVLHVDFDCLFEKGKRLPVPEIVPFRLTPNLLDALGI      2274
ATR       MVGYILGLGDRHGENILFDSLTGECVHVDFNCLFNKGETFEVPEIVPFRLTHNMVNGMGP      2525
ATM       IVGYILGLGDRHVQNILINEQSAELVHIDLGVAFEQGKILPTPETVPFRLTRDIVDGMGI      2920
TEL1      IVGYILGLGDRHLNNILLDCSTGEPIHIDLGIAFDQGKLLPIPELVPFRLTRDIVDGFGV      2662
          :**:******** :***::  :.: :*:*:.  *::*: :  ** ****** ::::.:*

MEC1      IGTEGTFKKSSEVTLALMRKNEVALMNVIETIMYDRNMDHSIQ-----------------      2317      G2279K
ATR       MGTEGLFRRACEVTMRLMRDQREPLMSVLKTFLHDPLVEWSKPVKGHSKAPLNETGEVVN      2585
```

```
ATM        TGVEGVFRRCCEKTMEVMRNSQETLLTIVEVLLYDPLFDWTMNPLKALYLQQRPEDETEL      2980
TEL1       TGVDGLFRRSCERVYAVLRKDYVKVMCVLNILKWDPLYSWVMSPVKKYEHLFEEEHEITN      2722
            *.:* *::..* .  ::*..   :: ::: :  *   .
MEC1       ---------------------------KALKVLRNKIRGIDPQDGLVLSVAGQTETLIQE      2350    S2339N
ATR        -------------------EKAKTHVLDIEQRLQGVIKTRNRVTGLPLSIEGHVHYLIQE      2626
ATM        HPTLNADDQECKRNLSDIDQSFNKVAERVLMRLQEKLKGV--EEGTVLSVGGQVNLLIQQ      3038
TEL1       FDN-------VSKFISNNDRNENQESYRALKGVEEKL------MGNGLSVESSVQDLIQQ      2769
                                           :.  :       *  **: . .. ***:
MEC1       ATSEDNLSKMYIGWLPFW 2368
ATR        ATDENLLCQMYLGWTPYM 2644
ATM        AIDPKNLSRLFPGWKAWV 3056
TEL1       ATDPSNLSVIYMGWSPFY 2787
           * . . *. :: **  :
```
